# Dynamic substrate topographies drive actin- and vimentin-mediated nuclear mechanoprotection events in human fibroblasts

**DOI:** 10.1186/s12915-025-02199-7

**Published:** 2025-04-07

**Authors:** Maaike Bril, Jules N. Boesveld, Leila S. Coelho-Rato, Cecilia M. Sahlgren, Carlijn V. C. Bouten, Nicholas A. Kurniawan

**Affiliations:** 1 Department of Biomedical Engineering, Eindhoven University of Technology, PO Box 513, 5600 MB Eindhoven, The Netherlands; 2 Institute for Complex Molecular Systems, Eindhoven University of Technology, PO Box 513, 5600 MB Eindhoven, The Netherlands; 3 Faculty of Science and Engineering, Åbo Akademi University, 20520 Turku, Finland

**Keywords:** Nucleus, Mechanotransduction, Dynamic topography, Histone modifications, DNA damage

## Abstract

**Background:**

Dynamic physical changes in the extracellular environment of living tissues present a mechanical challenge for resident cells that can lead to damage to the nucleus, genome, and DNA. Recent studies have started to uncover nuclear mechanoprotection mechanisms that prevent excessive mechanical deformations of the nucleus. Here, we hypothesized that dynamic topographical changes in the cellular environment can be mechanically transmitted to the nucleus and trigger nuclear mechanoprotection events. We tested this using a photoresponsive hydrogel whose surface topography can be reversibly changed on demand upon light illumination, allowing us to subject cells to recurring microscale topographical changes.

**Results:**

With each recurring topographical change, fibroblasts were found to increasingly compact and relocate their nuclei away from the dynamic regions of the hydrogel. These cell-scale reorganization events were accompanied by an increase of global histone acetylation and decreased methylation in cells on the dynamic topographies, resulting in a minimization of DNA strand breakage. We further found that these nuclear mechanoprotection events were mediated by both vimentin intermediate filaments and the actin cytoskeleton.

**Conclusions:**

Together, these data reveal that fibroblasts actively protect their nuclei in the presence of dynamic topographical changes through cytoskeleton-mediated mechanisms. Broadly, these results stress the importance of gaining a deeper fundamental understanding of the cellular mechanoresponse under dynamically changing conditions.

**Supplementary Information:**

The online version contains supplementary material available at 10.1186/s12915-025-02199-7.

## Background

It is increasingly recognized that living cells are mechanical bodies, whereby intra-, inter-, and extracellular mechanical forces are transmitted through the cytoplasm to the cytoskeleton and organelles. The mechanical signals can be converted into intracellular biochemical events in a process called mechanotransduction [1], thereby modulating downstream signaling events [2]. While such cellular signaling processes are normally slow (hours to days) [3], mechanical signal transmission can also be conducted via a variety of interconnected cellular cytoskeletal structures, leading to their physical deformation and sometimes alteration of their functions [4]. As a consequence, this mechano-structural pathway elicits a faster downstream response (minutes) [5]. A prime example of cellular mechanoresponsiveness is how culturing mesenchymal stem cells on micropillar arrays results in changes in nuclear morphology and chromatin organization [6]. The Linker of Nucleoskeleton and Cytoskeleton (LINC) complex, which physically connects the nuclear membrane, lamina, and the underlying chromatin with cytoskeletal polymers [7], allows direct mechanical connection between the nucleus and the surrounding extracellular matrix (ECM), leading to changes in nuclear morphology, chromatin organization, and the up- or downregulation of chemical residues on histones—so-called epigenetic modifications [6]. Such epigenetic modifications have also been shown to be induced by other extracellular mechanical cues, such as long-term culture of mesenchymal stem cells on stiff hydrogels, leading to persistent chromatin organization and stimulating osteogenic differentiation [8].

While this mechanical connection between the cell body and its extracellular environment is useful for rapid mechanosensing and mechanoresponse [9], it also exposes cells to potentially hazardous mechanical deformation and damage to critical organelles, including the nucleus. Indeed, nuclear abnormalities have been found in a range of human pathologies associated with defects of the nuclear envelope proteins. For example, mutations in nuclear lamina proteins cause nuclear fragility in mechanically active tissues, leading to nuclear damage and often cell death and premature mortality [7, 10]. In embryonic chick hearts, interference of actomyosin contractility and lamin A levels were found to drive nuclear rupture and the mislocalization of DNA repair factors, resulting in DNA damage and cell cycle arrest [11]. Additionally, confined cell growth can cause nuclear deformation, eventually leading to nuclear envelope rupture and DNA damage, promoting cell senescence in breast epithelial cells, but driving cancer metastasis in breast carcinoma [12]. Furthermore, cancerous cells show nucleus rupture and DNA damage during migration in confining microenvironments [13, 14]. Mechanical deformation of the nucleus itself, independent of nuclear envelope rupture, can already induce DNA damage and genome instability, contributing to tumorigenesis [15].

Interestingly, recent findings suggest that cells can actively protect their nucleus from mechanical perturbations, thus helping maintain genome integrity. For example, exposing epithelial cell layers to mechanical stretch results in nuclear wrinkling, decreased histone methylation, reduced lamina-associated heterochromatin, and lower nuclear membrane tension, altogether preventing DNA damage [16]. Moreover, prolonged exposure to cyclic stretch leads to cell alignment perpendicular to the stretch, lowering force transmission to the nucleus and reducing torsion and DNA breaking [16, 17].

Although it is clear that nuclear mechanoprotection events are vital to prevent nuclear rupture and DNA damage—and hence are essential to maintain tissue homeostasis, most studies so far have been performed in static or continuous stretch settings. These belie the fact that living, mechanically active tissues present dynamic extracellular environments and spatiotemporal topography changes to cells during development, aging, and disease progression [18]. For example, the naturally anisotropic microscale organization of the dermal ECM becomes increasingly anisotropic with maturation, but loses such anisotropy during wound healing, skin diseases, or scarring, thereby influencing cell phenotype, such as proliferation and migration [19–21]. The myocardium also has an anisotropic microscale ECM organization, which is not only critical for proper heart beating but also undergoes continuous geometrical changes due to the beating itself [22, 23], thereby subjecting the resident cells to dynamic topographical changes. Whether and how these dynamic topographical changes affect the resident cells and their nuclei remains unknown. Recently, we introduced a hydrogel-based cell culture platform to subject cells to dynamic topographies [24]. Here, we employed this approach to test the hypothesis whether dynamic topographies drive a mechanoprotective mechanism to protect the nucleus against mechanical perturbations. We observed that human fibroblasts, in the presence of recurring topographical changes, exhibit clear nuclear mechanoprotection signatures, including nuclear relocation from the dynamic regions, nuclear shape irregularities, and altered histone acetylation and methylation levels. Moreover, our data indicate that this mechanoprotection relies on both the integrity of the actin cytoskeleton and the presence of vimentin intermediate filaments.

## Results

### Dynamic topographies change nucleus morphology and location

**Fig. 1:**
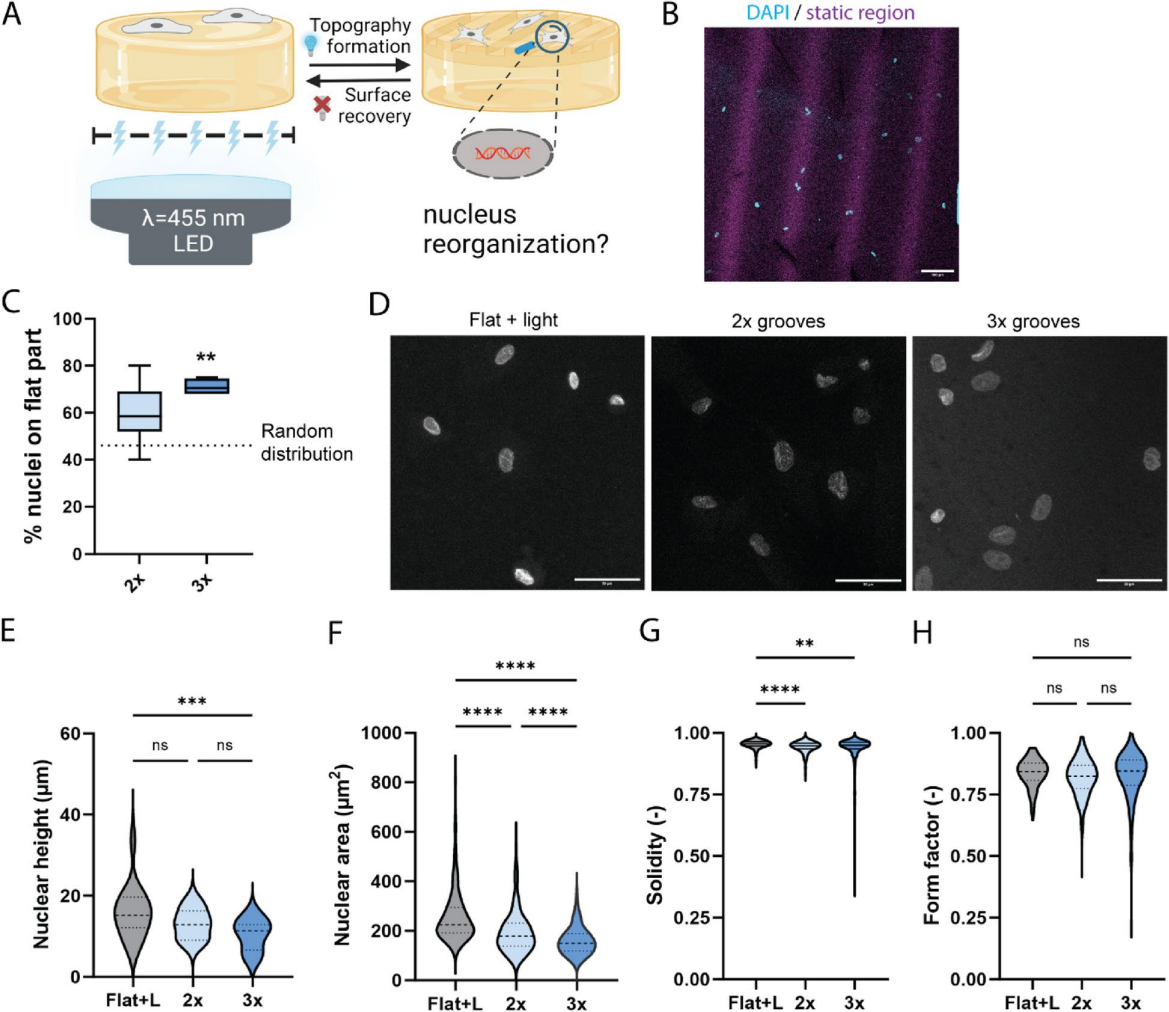
Dynamic topographies affect nucleus morphology. **A** Schematic of the experiment to examine the influence of dynamic topographies on nucleus morphology. **B** Representative image of nucleus positioning of human dermal fibroblasts on the dynamic gel after 3 rounds of topographical changes (90 µm-wide grooves). Blue: DAPI-stained nuclei, purple: flat, static regions of the hydrogel. Scale bar is 100 µm. **C** Quantification of fraction (percentage) of nucleus located on the flat (static) part of the hydrogel. Data are represented in a box-and-whiskers plot showing the median, minimum value, maximum value, and the 25^th^ and 75^th^ percentiles. The dotted line indicates a random distribution of the nuclei across flat and dynamic regions. One-way ANOVA with Tukey’s multiple comparison, *p* = 0.0029, compared to a random distribution of nuclei. *n* = 4 hydrogels with at least 2 processed field of views per gel and ≥ 22 cells per field of view. **D** Representative images of human dermal fibroblast nuclei (DAPI) with the different topography actuation protocols (2 × or 3 × repeated topographical changes from flat to 90 µm-wide grooves). Scale bar is 50 µm. **E** Nucleus height after 2 × or 3 × rounds of topographical changes (flat to 90 µm-wide grooves). Data are shown in violin plots with dashed lines indicating the median and dotted lines indicating the quartiles. Kruskal–Wallis test with Dunn’s multiple comparisons, *** *p* = 0.0004, *n* ≥ 27 randomly selected nuclei of fibroblasts cultured on dynamic topographies (2 × or 3 × rounds). **F**–**H** Nucleus area, solidity, and form factor after 2 × or 3 × rounds of topographical changes (flat to 90 µm-wide grooves). Data are represented in violin plots, with dashed lines indicating the median and dotted lines indicating the quartiles. Flat + L = flat gel (not photoresponsive) with light exposure (control). Kruskal–Wallis test with Dunn’s multiple comparison test with **** *p* < 0.0001, *** *p* < 0*.*001, ** *p* < 0.01, *n* ≥ 197 cells per condition

In our previous work, we developed a photoresponsive, cell-compatible spiropyran-based hydrogel that reversibly changes its surface topography within 15 min upon local blue light (455 nm) exposure, resulting in topographies of user-defined microscale geometries and dimensions with negligible alteration of other properties of the hydrogel that may be sensed by cells, including stiffness, surface roughness, and stretch [24]. Removal of the light reverts the hydrogel to its original flat surface, allowing us to subject cells to multiple rounds of recurring and/or reconfiguring topographies. Microscale topographies were selected to mimic the typical anisotropic topographical cues present in the ECM of living tissue such as skin, heart, muscle, and tendon [25–27]. Furthermore, the chosen dimensions fall within the range of small vasculature (e.g., alveoli with diameters of 75–300 μm), gut villi, and small wounds (nano to millimeter scale) [25, 28]. Previously, the creation and disappearance of groove or pit topographies (50–150 µm wide) were found to induce reorganization of cell shape and regulate focal adhesion formation as well as nuclear localization [24]. Here, we used this light-responsive shape-morphing hydrogel as a substrate for culturing fibroblasts and examined the effect of dynamic topographies on mechanotransduction to the nucleus. We subjected the cells to multiple rounds of changing topographies (2 × 3 h), alternating between flat and grooves of 90 µm width and 350 nm height (Fig. 1A; Additional file 1: Fig. S1). In line with our previous finding [24], we observed that the cell nuclei were frequently found away from the dynamic regions (Fig. 1B). Remarkably, this nuclear positioning away from the dynamic regions was not accompanied by selective detachment of cells from the dynamic regions nor any significant migration of cells within the experimental timeframe, strongly suggesting that the cells intracellularly relocated their nuclei. Since nuclear relocation was induced by the dynamic change in substrate topography, we asked whether additional rounds of topography change would lead to more increased occurrences of nuclei relocation. Indeed, we found increased nuclear relocation upon more rounds of actuation while keeping the total culture time unchanged (3 × 2 h), reaching 71 ± 3.6% of all nuclei being on the flat, static regions after 3 rounds of topography actuation (Fig. 1C), again corroborating nuclear relocation by the cells. As intracellular movement of cellular organelles through the confined cytoplasmic space often results in organelle deformation [29], nuclear morphology can be used as an indicator for active nuclear relocation. Consistent with this finding, we observed changes in the nuclear morphology after the topography actuations (Fig. 1D). Quantification of nuclear shape parameters revealed that additional rounds of topographical changes resulted in significantly smaller nuclei (~ 40% reduction of nuclear area, *p* < 0.0001) at unchanged nuclear heights (Fig. 1E, F). The nuclei displayed more irregular shapes (in 2D), as reflected in the lower solidity values (Fig. 1G, p < 0.0001 (2 × conditions), *p* = 0.0027 (3 × conditions)). The form factor—a measure of nuclear circularity—did not differ between the experimental groups; however, the distribution of values broadened significantly with more rounds of topographical changes (Fig. 1H), in line with the notion that the topographical changes induced cells to relocate and consequently deform their nuclei.

### Dynamic topographies induce epigenetic changes to prevent DNA damage

**Fig. 2:**
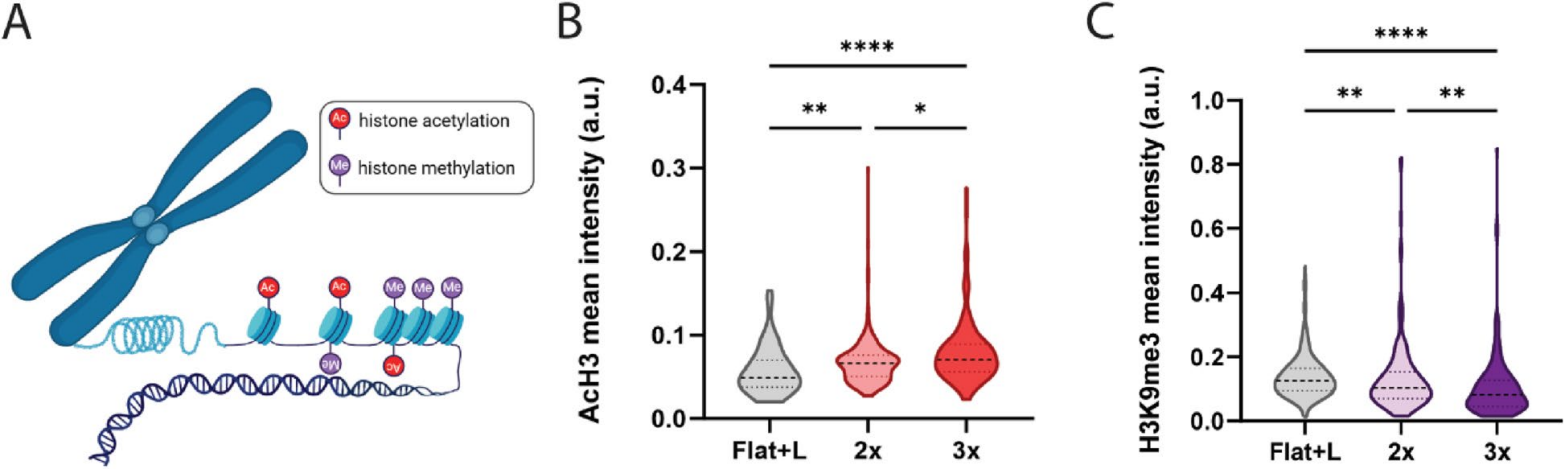
The presence of dynamic topographies changes the epigenome. **A** Schematic representation of epigenetic modifications on histone tails. **B** Quantification of mean histone acetylation (AcH3) intensity levels in response to dynamic topographies (2 × or 3 × 90 µm-wide grooves) or flat + light (Flat + L) control conditions; a.u. arbitrary units. *n* ≥ 91 cells per condition. **C** Quantification of mean histone trimethylation (H3K9me3) intensity levels in response to dynamic topographies (2 × or 3 × 90 µm-wide grooves). *n* ≥ 126 cells per condition. Kruskal–Wallis test with Dunn’s multiple comparison test with **** *p* < 0.0001, *** *p* < 0*.*001, ** *p* < 0.01, * *p* ≤ 0.05. Data are represented in violin plots, with dashed lines indicating the median and dotted lines indicating the quartiles

Mechanical cues from the cellular microenvironment have been shown to impact the chromatin architecture and epigenome. For example, stiffness [8, 30] and microtopographies [6, 31] affect histone methylation and acetylation levels in fibroblasts and stem cells. Moreover, mechanical stretching of epithelial monolayers induced a decrease in histone methylation levels and heterochromatin [16]. As we observed changes in the nuclear morphology with topography actuation, we hypothesized that the nuclear deformation may also affect epigenetic regulation, specifically the presence of chemical groups (e.g., acetyl or methyl) on histone tails that thereby influence chromatin folding (Fig. 2A). Indeed, we found that histone acetylation (AcH3) levels increased with more rounds of changing topographies (16% for 2 × (*p* = 0.0111) and 40% for 3 × conditions (*p* < 0.0001)) (Fig. 2B and Additional file 1: Fig. S2). Histone trimethylation (H3K9me3) levels only showed a minor reduction between flat and 2 × rounds of topography changes (4%, *p* = 0.0483), while an additional round of topographical changes (3 ×) decreased the intensity levels more (ca. 20%, *p* < 0.0001) (Fig. 2C and Additional file 1: Fig. S3). The epigenetic regulation may be attributable to dynamic topography-induced mechanotransduction via an enhanced nuclear localization of mechanosensitive transcription factors such as YAP/TAZ [6, 8]. To gain a better understanding of how the presence of dynamic topographical cues acts on the epigenome, fibroblasts were subjected to changing topographies and stained for YAP/TAZ. Unexpectedly, the presence of dynamic topographies did not influence YAP/TAZ nuclear-to-cytoplasm localization ratio (Additional file 1: Fig. S4). Although the observed epigenetic response to dynamic topographies was not mediated by altered YAP/TAZ activity, other mechanosensitive transcription factors, such as MRTF-A [32], may still be involved.

**Fig. 3:**
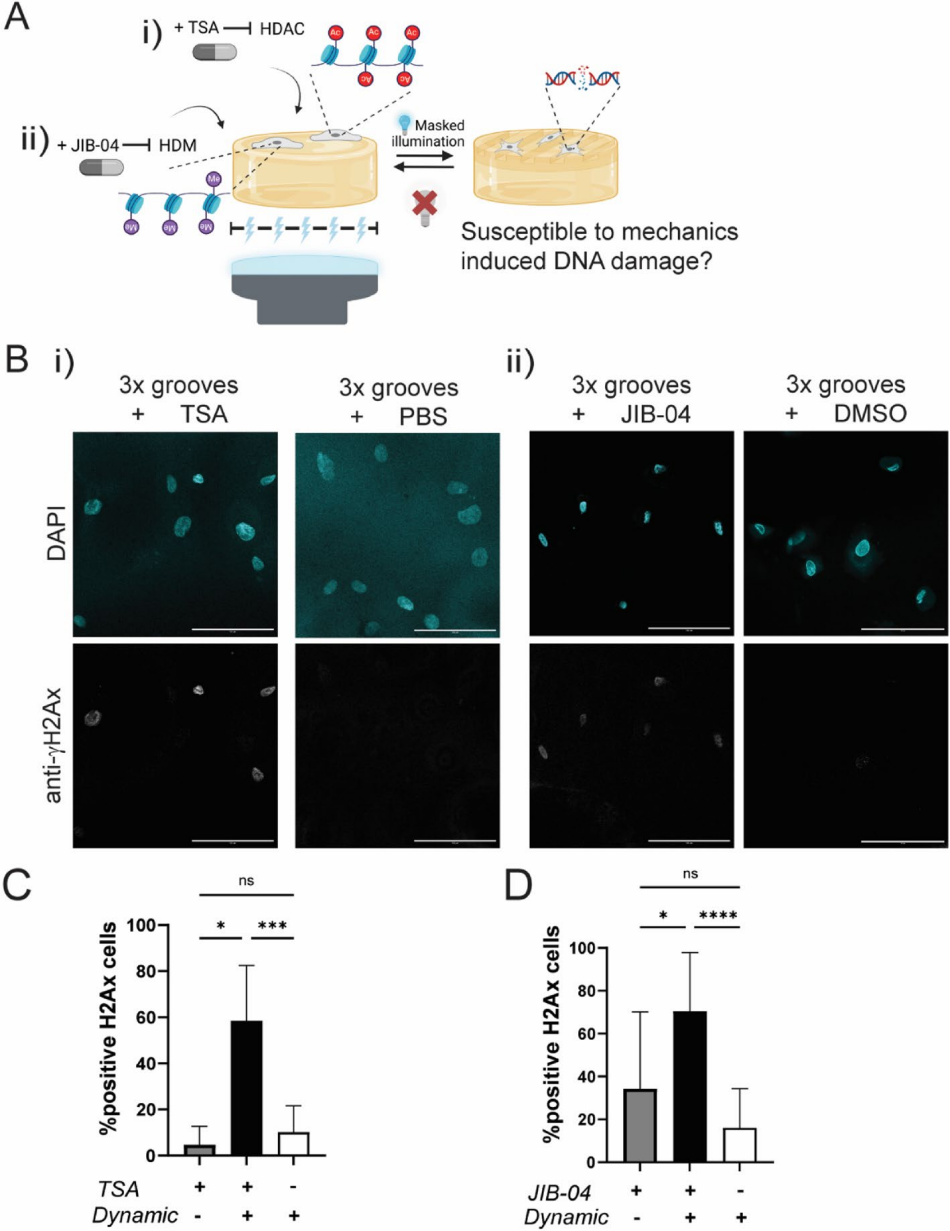
Epigenetic modifications help to prevent DNA damage during dynamic topographical changes (3 × grooves). **A** Illustration of the cell culture experiment to investigate the link between the epigenome and DNA damage, by adding TSA, a histone deacetylase (HDAC) inhibitor (i) or by adding JIB-04 (ii), a histone demethylase (HDM) inhibitor. **B** (i) Representative images of nuclei treated with 0.5 µM TSA or PBS. Blue, DAPI, gray, γH2Ax (marker for double-stranded DNA breaks). Scale bar: 100 µm. (ii) Representative images of nuclei treated with 10 µM JIB-04 or DMSO. Blue, DAPI, gray, γH2Ax. Scale bar is 100 µm. **C** Quantification of γH2Ax-positive nuclei treated with 0.5 µM TSA or no TSA (PBS only), cultured on flat or dynamic surface topographies. Kruskal–Wallis test with * *p* = 0.0149, *** *p* = 0.0002, ns *p* > 0.9999 with *n* ≥ 108 cells (2 independent experiments). **D** Quantification of γH2Ax-positive nuclei treated with 10 µM JIB-04 or no JIB-04 (DMSO only), cultured on flat or dynamic surface topographies. Kruskal–Wallis test with * *p* = 0.0158, **** *p* < 0.0001, ns *p* = 0.05785, with *n* ≥ 64 cells (3 independent experiments). Data are shown as mean ± SD

At first glance, the increase in AcH3 levels upon topography actuation appeared counterintuitive, since the presence of these negatively charged groups should theoretically result in a more open chromatin state and therefore larger nuclei, which is the opposite of our observation. A delicate balance in epigenetic modifications is required to maintain chromatin organization, but also to ensure DNA repair, since a more open chromatin organization is more accessible to DNA repair factors [33]. In other words, it is conceivable that the mechanoresponsive changes in AcH3 levels are needed to indirectly protect the DNA against mechanically induced damage (Fig. 3A). To test this, fibroblasts were treated with trichostatin A (TSA), a drug that inhibits histone deacetylase (HDAC) activity and thereby creates an even more open chromatin organization due to increased acetylation levels [34]. TSA was added 30 min before the first topographical change, and fibroblasts were subsequently subjected to 3 rounds of topographical actuations (Fig. 3Ai; Additional file 1: Fig. S5). The presence of γH2AX foci, a marker for double-stranded DNA breaks [33, 35], was checked using confocal microscopy. TSA-treated cells cultured on flat substrates showed a small percentage of γH2AX-positive cells (5%), as expected [36]. Also consistent with our expectation, TSA-treated fibroblasts on dynamic topographies showed significantly more DNA damage than vehicle-treated fibroblasts and TSA-treated fibroblasts on flat, illuminated gels (Fig. 3Bi, C; Additional file 1: Fig. S6). Moreover, TSA-treated fibroblasts displayed nuclear wrinkling and shape irregularities (Fig. 3B). The data confirms that dynamic topographies provoke a nuclear mechanoresponse, resulting in higher AcH3 levels to allow DNA repair factors to access the chromatin, which ultimately helps to protect the genome against mechanics-induced damage.

Furthermore, we hypothesized that H3K9me3 modifications might serve a similar role as AcH3 in preventing DNA damage. Therefore, fibroblasts were seeded on the hydrogels and 30 min before actuation JIB-04 was added to the medium to inhibit histone demethylase (HDM) activity and to interfere with the chromatin organization [37] (Fig. 3Aii; Additional file 1: Fig. S7). JIB-04 treatment of fibroblasts on flat substrates resulted in γH2AX-positive cells. Interference of histone methylation levels also resulted in the formation of γH2AX foci in response to dynamic topographies, which was abrogated in vehicle-treated fibroblasts on dynamic topographies (Fig. 3Bii, D; Additional file 1: Fig. S6). Altogether, these findings indicate that the presence of epigenetic modifications (AcH3 and H3K9me3) assists the repair of DNA breaks in fibroblasts as triggered by dynamic topographies.

### Vimentin intermediate filaments and actin cytoskeleton are crucial drivers of nuclear mechanoprotection and provide a failsafe mechanism

**Fig. 4:**
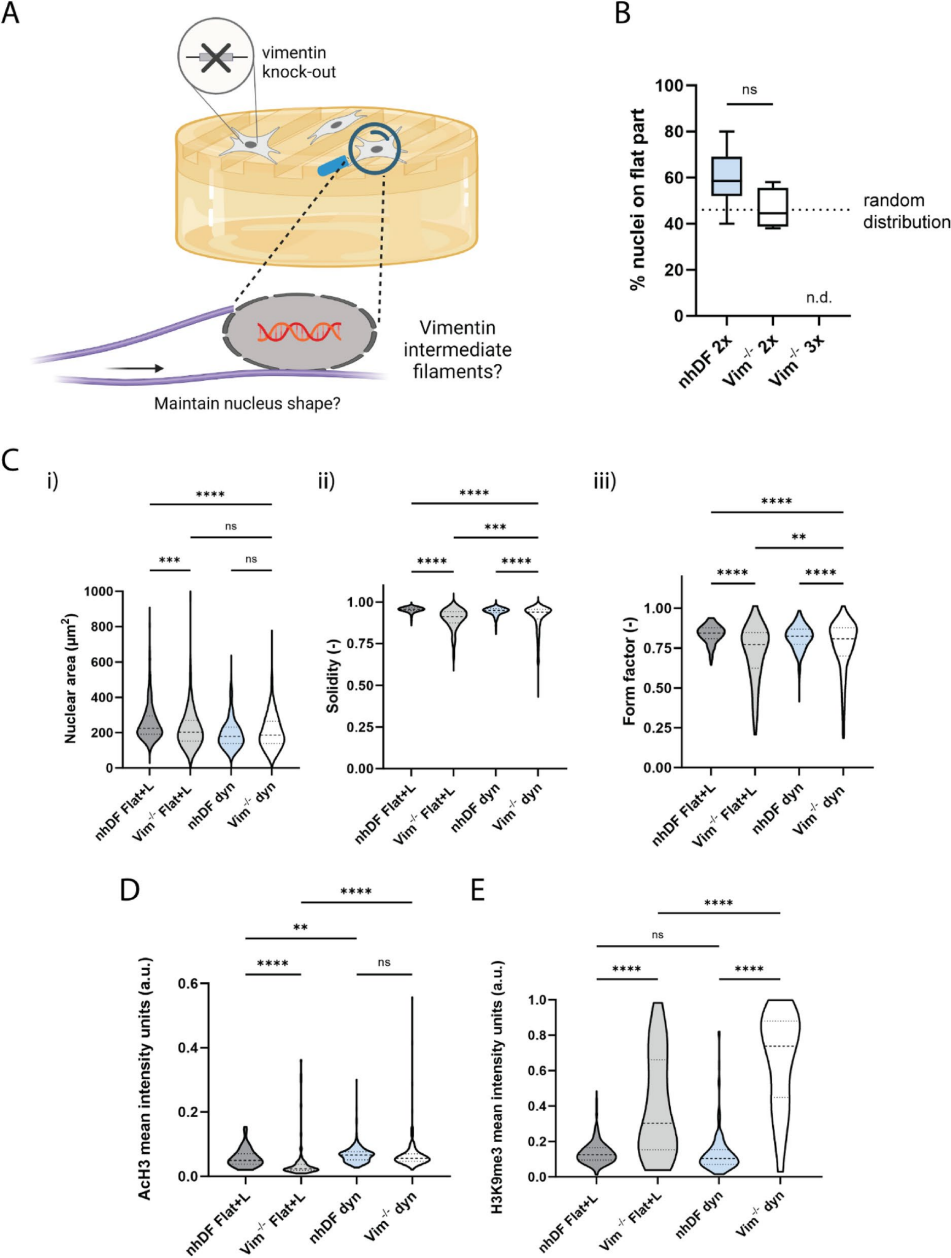
Vimentin intermediate filaments are required for nuclear mechanoprotection in a dynamic environment. **A** Illustration of the hypothesis that vimentin intermediate filaments are important as a keeper for nucleus shape. **B** Quantification of the percentage of normal human dermal fibroblasts (nhDF) or Vim^−/−^ fibroblast nuclei located on the flat regions of the hydrogel, n.d. = not detectable, all cells detached after 3 rounds of surface actuation. Dotted line indicates a random distribution of the nuclei across flat and dynamic regions. Data are represented in a box-and-whiskers plot showing the median, minimum value, maximum value, and the 25th and 75th percentiles. One-way ANOVA with Tukey’s multiple comparison, *n* = 2 hydrogels with at least 2 processed field of views per gel and 221 cells in total. **C** Quantification of nucleus morphology parameters after 2 rounds of surface actuation: nucleus area (i), solidity (ii), and form factor (iii), *n* ≥ 303 cells/condition. Dyn = cells on dynamic hydrogels (2 × grooves), Flat + L = cells on flat gels with light exposure. **D** Mean AcH3 expression (a.u.) in Vim^−/−^ fibroblasts on dynamic hydrogels (2 × grooves, dyn) or on flat gels with light exposure (Flat + L), *n* ≥ 77 cells/condition. **E** Mean H3K9me3 expression (a.u.) in Vim^−/−^ fibroblasts on dynamic hydrogels (2 × grooves, dyn), *n* ≥ 96 cells/condition. Data in **C**–**E** are represented in violin plots, with dashed lines indicating the median and dotted lines indicating the quartiles. Kruskal–Wallis test with Dunn’s multiple comparison test with **** *p* < 0.0001, ** *p* = 0.0028

Next, we sought to gain a better understanding of how fibroblasts are capable of driving the observed nuclear mechanoprotection events. Based on previous studies reporting on the role of cytoskeletal tension on nucleus morphology and chromatin remodeling [8, 30], we reasoned that the direct transmission of mechanical forces, originating from the dynamic topography, to the nucleus via the cytoskeleton, might influence the epigenome directly. Furthermore, the actin cytoskeleton and intermediate filaments have been shown to play important roles in cell mechanics and intracellular mechanical force generation [38–40]. Both cytoskeletal polymers are coupled to the LINC complex and allow force transmission from the extracellular matrix (ECM) to the LINC complex and across it, providing mechanical access to the regulation of chromatin organization. In addition, actin integrity allows mechanotransduction and force transmission and intermediate filaments are known for their load-bearing capacity and keepers of cell and organelle shape [39, 41, 42]. Interestingly, a distinct vimentin expression pattern in fibroblasts on dynamic topographies can be seen in Additional file 1: Fig. S8; the classic perinuclear cage is absent and fibroblasts display a “nutshell” distribution of vimentin. To investigate the contribution of vimentin intermediate filaments on nuclear mechanoprotection in more detail, we exposed vimentin-knock-out (Vim^−/−^) immortalized fibroblasts to dynamic topographies (Additional file 1: Fig. S9 and Additional file 2: Fig. S1). The immortalized wildtype fibroblasts showed a similar nuclear mechanoprotection response as the normal fibroblasts after exposure to 3 rounds of dynamic topographies (Additional file 1: Figs. S10 and S11). After 3 rounds of dynamic topographies (2 h per round), all Vim^−/−^ cells detached, providing a first indication that vimentin filaments play a role in the mechanoresponse to dynamic topographies (Fig. 4A). Strikingly, 2 rounds of dynamic topographies (3 h per round) revealed that Vim^−/−^ fibroblasts were unable to relocate their nuclei away from the dynamic topographical regions (Fig. 4B). Moreover, Vim^−/−^ fibroblast nuclei showed a similar nuclear compaction as normal fibroblasts, with more pronounced shape irregularities on dynamic topographies (Fig. 4C). In terms of the resulting epigenetic regulations, Vim^−/−^ fibroblasts displayed equal AcH3 levels as the normal fibroblasts, but their H3K9me3 levels were significantly higher than those of the fibroblasts on dynamic topographies (Fig. 4D, E; Additional file 1: Fig. S12).

**Fig. 5:**
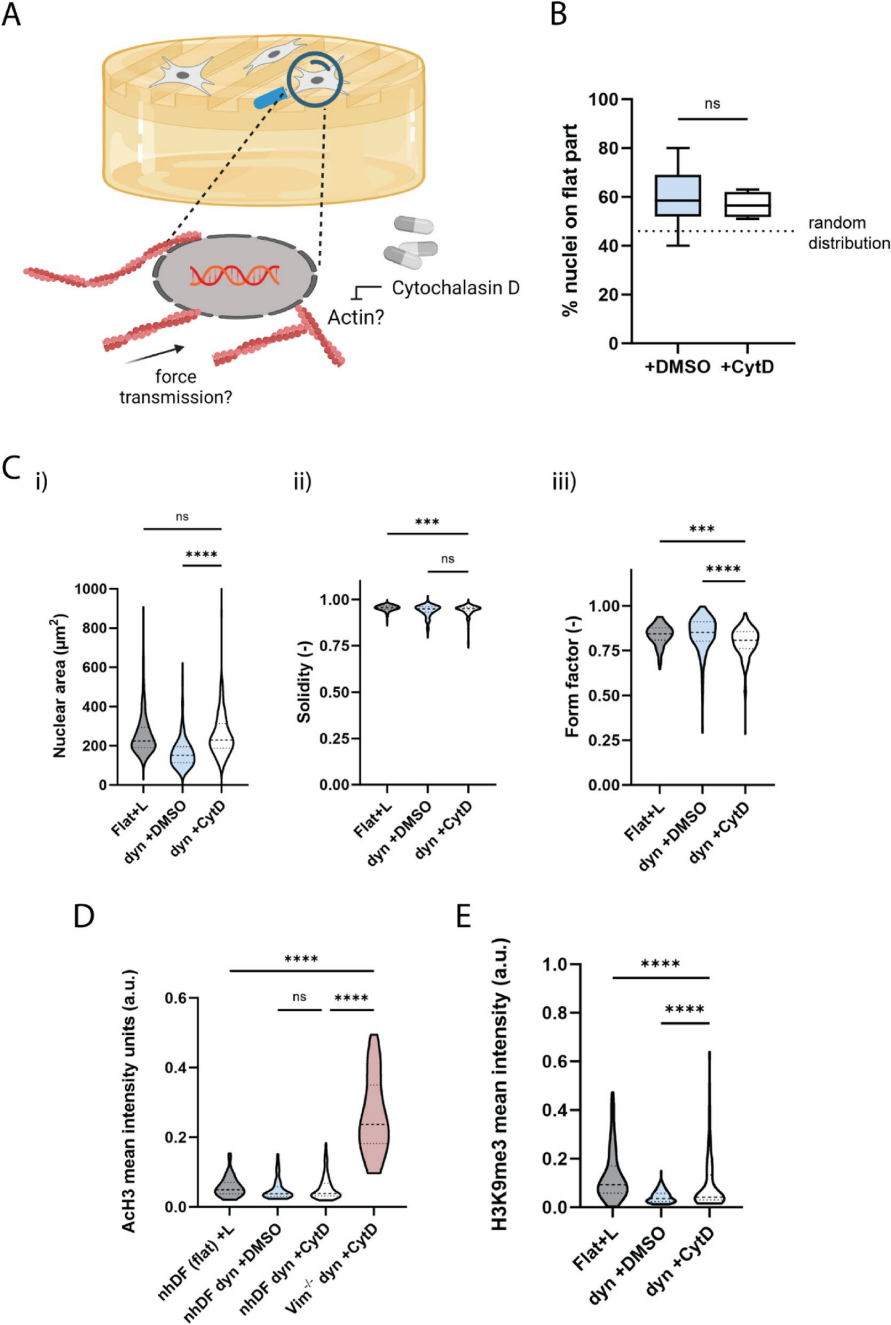
Actin cytoskeleton polymers are involved in nuclear mechanoprotection in dynamic environments. **A** Illustration of the hypothesis that actin fibers are important regulators of nuclear mechanoprotection. **B** Quantification of CytD-treated fibroblast nuclei on the flat part of the hydrogel. One-way ANOVA with Tukey’s multiple comparison, *n* = 2 hydrogels with at least 2 processed field of views per gel and 151 cells in total. Dotted line indicates a random distribution of the nuclei across flat and dynamic regions. Data are represented in a box-and-whiskers plot showing the median, minimum value, maximum value, and the 25th and 75th percentiles. **C** Quantification of nucleus morphology parameters: nucleus area (i), solidity (ii), and form factor (iii), with *n* ≥ 108 cells/condition. Flat + L = flat gels with light exposure. **D** Mean AcH3 expression (a.u.) in CytD-treated fibroblasts on dynamic (dyn) hydrogels (2 × grooves), with *n* ≥ 53 cells/condition. nhDF = normal human dermal fibroblast, Vim^−/−^ = vimentin-knockout fibroblast. **E** Mean H3K9me3 expression (a.u.) in CytD-treated fibroblasts on dynamic hydrogels (2 × grooves), with *n* ≥ 70 cells/condition. Data in **C**–**E** are represented in violin plots, with dashed lines indicating the median and dotted lines indicating the quartiles. Kruskal–Wallis test with Dunn’s multiple comparison test with **** *p* < 0.0001, *** *p* < 0.001

The polymerization and dynamics of actin filaments is crucial not only for mechanotransduction, but also for mechanical force transmission and generation, which may lead to translocation and deformation of organelles [43, 44]. To examine the role of actin integrity in the nuclear mechanoresponse, fibroblasts were treated with cytochalasin D (CytD), which inhibits actin polymerization, and exposed to dynamic topographies (2 rounds) (Fig. 5A). The result shows that actin was also required to relocate the nuclei away from dynamic regions (Fig. 5B), but this response was notably less pronounced than the contribution of vimentin filaments in nuclear relocation (Fig. 4B). Also, in contrast with what was observed for Vim^−/−^ fibroblasts, CytD-treated fibroblast nuclei showed no nuclear compaction in response to dynamic topographies, and the nucleus shape remained unaffected (Fig. 5C). Interestingly, AcH3 levels were not elevated in CytD-treated fibroblasts in response to dynamic topographies (Fig. 5D; Additional file 1: Fig. S13). Moreover, H3K9me3 levels of CytD-treated fibroblasts also increased, though to a lesser extent than Vim^−/−^ fibroblasts in response to dynamic topographies (Figs. 5E and 4E; Additional file 1: Fig. S14).

These findings indicate that vimentin and actin cytoskeleton are both inherently involved, but have distinct roles in regulating the cell’s nuclear shape and the associated epigenetic changes in response to dynamic topographies. We therefore questioned whether this represents a redundant nuclear mechanoprotection mechanism whereby the interplay between vimentin and actin and their epigenetic targets allows to preserve a nuclear mechanoprotection mechanism to collectively ensure minimal nuclear damage from topographical perturbances. Both cytoskeletal polymers can transmit extracellular forces to the nucleus, thereby allowing the cell to adapt its AcH3 and H3K9me3 levels in the presence of dynamic topographies. If one cytoskeletal component fails, the other component will take over, explaining the similar outcome in AcH3 and H3K9me3 levels upon disturbance with one cytoskeletal component in response to dynamic topographies. If this is true, dual disruption of both vimentin and actin cytoskeletal polymers should increase AcH3 levels. To test this hypothesis, Vim^−/−^ fibroblasts were treated with cytochalasin D and subjected to dynamic topographies (2 rounds). Indeed, AcH3 levels in CytD-treated Vim^−/−^ fibroblasts were strongly elevated on dynamic topographies, much beyond those of the controls, confirming the synergistic role of actin and intermediate cytoskeletal polymers in eliciting an epigenetic-based nuclear mechanoprotection response (Fig. 5D).

## Discussion

In living tissue, cells are exposed to dynamic microenvironments, for example, during tissue homeostasis, disease progression*,* wound healing, and aging. These mechanical perturbations might damage the nucleus and genome, and thus, cells require mechanisms to prevent and repair mechanically induced DNA damage. For example, a disturbed nuclear mechanosensing is associated with myofibroblast persistence and excessive chromatin condensation, ultimately leading to fibrosis [30]. Although research in the last decade established the importance of the nucleus as a mechanoresponsive organelle, it is far from well understood how cells prevent unwanted chromatin reorganizations and DNA damage upon mechanically induced nuclear deformation. Besides, the influence of the highly dynamic nature of the ECM on nuclear mechanoresponsiveness is another aspect that is often overlooked.

Here, we use recently established shape-morphing photoresponsive hydrogels [24] to mimic the dynamic ECM and demonstrate that dynamic topographies drive a nuclear mechanoprotection mechanism in fibroblasts. In the presence of dynamic topographies (alternating between flat and grooves), both vimentin and actin cytoskeletal polymers were found to orchestrate mechanoprotection events, leading to nuclear compaction and relocation away from dynamic regions. Previous work already showed that nuclei can be deformed by the underlying (static) substrate topography. More specifically, when cultured on micropillars, hMSCs wrap their DNA around the micropillars and adjust the degree of heterochromatin [6], but that nuclear envelope integrity was maintained, probably to protect the genome. Static topographies have also been reported to induce changes in nucleus morphology, where both nanotopographies (250 nm gratings) and microtopographies (10 µm) were found to influence nucleus elongation, size, and morphology [6, 31, 45]. Our study translates these findings to dynamically changing topographies and demonstrates that fibroblast nuclei adapted on these dynamic topographies: the nuclei became smaller in size and exhibited irregular shapes. Notably structural elements of the nucleus, e.g., nuclear pore complexes, and/or the cytoplasm, e.g., perinuclear actin caps and intermediate filaments, regulate and influence nucleus size and shape, explaining the observed decrease in nucleus size despite the upregulation of epigenetic markers [46]. Furthermore, we show that fibroblasts move their nucleus away from the regions with changing topographies, consequently safeguarding their genetic material. This nuclear repositioning response shows for the first time that the microenvironment dynamics can crucially affect cell behavior by rapid mechano-structural signaling events, which could not have been found using traditional, static topographical cell-culture platforms. To further understand nucleus relocation and protection events in more detail, advanced live-cell imaging and visualization of motor-mediated actin forces should be exploited in future studies.

**Fig. 6:**
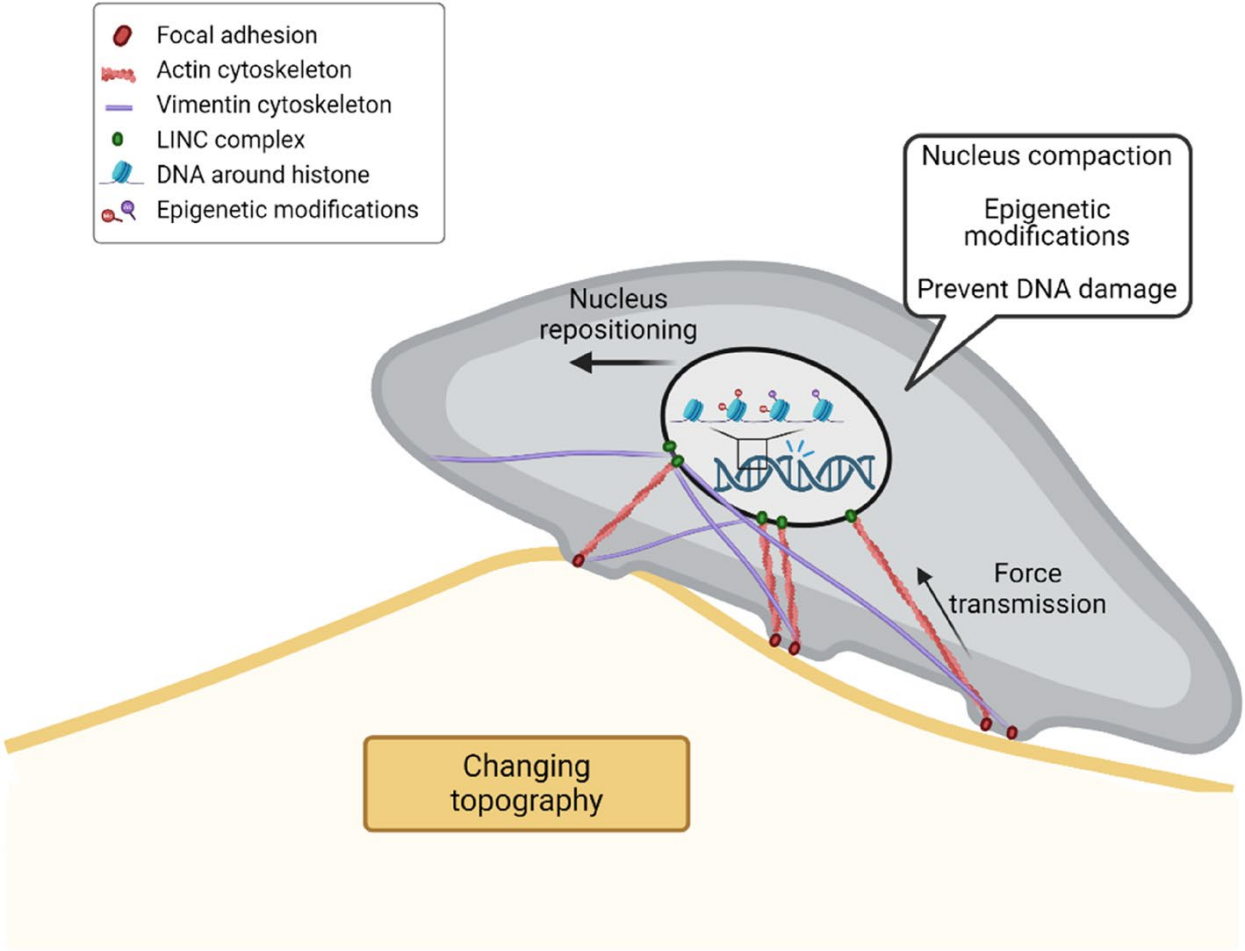
Cartoon summarizing the discovered nuclear mechanoprotection events that happen in response to dynamic topographies. Actin and vimentin intermediate filament networks transmit forces intracellularly, thereby activating a nucleus mechanoprotection mechanism. Fibroblasts compact their nuclei and relocate it away from dynamic regions. Additionally, up- and downregulation of epigenetic modifications help to prevent mechanics-induced DNA breaks

Both the actin cytoskeleton as well as vimentin intermediate filaments were found to be critical in the direct transmission of forces to the nucleus and for eliciting a nuclear mechanoresponse. The presence of perinuclear actin caps and intermediate filament networks allow the cell to mechanically shield its nucleus, prevent nuclear rupture, and preserve nuclear stability and integrity during mechanical perturbations [47–51]. The importance of both cytoskeletal networks in mechanoprotection has started to be explored in previous works [47, 52]. For example, transmission of forces via the actin cytoskeleton can deform the nucleus, especially via the presence of the perinuclear actin cap, leading to reorganization of the cytoskeleton and cell phenotype [53–55]. This actin cap displays a fast reorganization response in the presence of dynamic mechanical cues, such as fluid shear flow or uniaxial cyclic strain [40, 55, 56]. Additionally, a distinct vimentin subcortical distribution is observed in fibroblasts on dynamic topographies. This is in accordance with the data of Feliksiak et al. who observed a vimentin “nutshell” distribution instead of the classic perinuclear cage [57]. This subcortical vimentin configuration was found to control actin cortex organization and contractility during mitosis [58] and enables protection against nucleus misplacement in the cytoplasm during migration [41]. Very recently, keratin intermediate filaments were found to protect the nucleus from actin-mediated deformations by stiffening the keratin cytoskeleton and influencing chromatin methylation levels (H3K27me3), another marker for heterochromatin [49]. Both actin and vimentin intermediate filaments, and not microtubules [52], were found to regulate nuclear morphology and chromatin interactions in embryonic stem cells. Additionally, high-resolution microscopy and cryo-electron tomography showed that actin and vimentin have structural interactions within the cell cortex and form interpenetrating networks [59]. Crosslinks between actin and intermediate filaments, such as by the plectin protein family, may fulfill an important role in the observed nucleus response [60, 61]. Moreover, a recent study modeled vimentin interactions with microtubules and actin and revealed that vimentin contributes to local cellular force transmission and increases the range of force propagation [62]. Together, these studies stress the existence of cytoskeletal crosstalk, and thereby the possible cooperation between cytoskeletal networks to safeguard the nucleus. Our present study adds to this growing body of evidence by showing that the cytoskeletal-mediated transmission of extracellular forces induces a nuclear mechanoprotection response to prevent damage of the genome. This nuclear protection consists of several events: (1) nuclear compaction, driven by the actin cytoskeleton, and maintenance of nuclear shape, driven by vimentin filaments; (2) nuclear relocation away from dynamic regions; (3) change of AcH3 and H3K9me3 levels allowing DNA repair factors to access the chromatin, in case of mechanically induced damage (Fig. 6). Notably, while YAP/TAZ did not seem to be involved in the epigenetic regulation during dynamic topographical conditions, translocation of other (mechanosensitive) transcription factors, or the activation of other intracellular signaling components, such as kinases, receptors, or via posttranslational protein modifications [63–66], can also play a role in the regulation of epigenetic modifications and cannot be ruled out at this moment. Taken together, likely due to the interplay between both networks, force transmission to the nucleus can be strictly, specifically, and selectively regulated to discriminate between mechanoprotection and mechanoactivation events. It is conceivable that actomyosin contractility allows the initiation of the mechanoresponse, which is then sustained by vimentin intermediate filaments [67]. Furthermore, the interplay between actin and vimentin might provide the cell with a redundant, failsafe mechanism of nuclear force transmission, allowing to elicit an epigenetic response, even during failure of one cytoskeletal component. The exact mechanism by which mechanical cues and cytoskeletal forces can influence chromatin modifications and organization should be studied in more detail in the future.

Culture of fibroblasts on dynamic topographies revealed an increase of AcH3 levels, which is in line with a previous study of mouse fibroblasts on static 10 µm microgrooves [31]. Using our current set-up, H3K9me3 levels were downregulated in dermal fibroblasts. Interestingly, another study showed that the presence of nanogrooves (250 nm wide) increased H3K9me3 levels in human embryonic stem cells [45]. On the contrary, culture of human mesenchymal stem cells on micropatterns (5 µm grids) resulted in decreased H3K9me3 expression. These differences in H3K9me3 expression might be due to cell specific mechanosensitivity or the use of different cell types or dimensions of the topographies. Alternatively, they may also result from a fundamental difference between cell response to static or dynamic environments, which will be exciting to further probe. Recently, the height of topographical cues was found to influence fibroblast orientation and morphology [68]. It will be interesting to explore possible amplitude-dependent effects of dynamic topographies on nucleus mechanoresponse, for example, by varying the amount of crosslinker in our spiropyran-based hydrogel system, thereby allowing variations in the depth of the created dynamic structures.

Our work shows that dynamic topographies alter AcH3 and H3K9me3 levels probably to allow repair of DNA breaks. This mechanism enables the protection of genetic material against unwanted deformations and prevents damage of the genome. DNA breaks-induced histone modifications have been reported to mediate in detecting DNA damage, initiating repair processes, and facilitating DNA repair [69–71]. Our findings are in line with previous research, where epithelial tissues display chromatin reorganization to protect the genome from stretch-induced damage. Interestingly, chromatin reorganization is abolished when histone methylation levels are increased, leading to DNA damage [16]. It will be interesting in the future to check whether the topography-induced changes in the epigenome also contribute to altered chromatin architecture and reorganizations, by using, e.g., ATAC-sequencing, and to follow histone modification in real-time using advanced imaging set-ups. Furthermore, the concept of mechanical memory also gained increased attention, whereby a mechanical input can prime and regulate cell responses later in (culture) time by leaving a “mark” in the epigenome, which can be referred to as a mechanical memory [8, 72, 73]. Since changes in the epigenome can have a big impact on gene regulation and transcription, it can thereby influence cell fate [74]. Hence, this fundamental knowledge can contribute to a better understanding of cell behavior in the constantly dynamically changing ECM.

## Conclusions

In summary, we used a photoresponsive spiropyran-based hydrogel that allows us to change surface topography on demand to examine the effect of dynamic topographies on fibroblast nucleus behavior. Fibroblasts demonstrated a clear nuclear mechanoprotection response; nucleus morphology was adjusted, and nuclei were relocated away from the dynamic regions. Moreover, histone acetylation and methylation levels were altered to protect the genome from unwanted, mechanically induced DNA damage. For this, the mechano-structural signaling pathway is essential to directly transmit forces to the nucleus and to elicit a nuclear mechanoprotection mechanism.

## Methods

### Light-responsive hydrogel-based cell culture platform

SBS-coated spiropyran-containing poly(N-isopropylacrylamide) hydrogels were prepared as described previously [24] and placed in a custom-build illumination holder that fits a 12-well plate. Hydrogels were illuminated via the bottom through soda-lime photomasks with chromium-designed patterns (designed in KLayout v 0.26.9 and produced by Techno-Mask, Eindhoven). Light illumination was performed using collimated LEDs (455 nm, 700 mA, M455L4-C4, Thorlabs) connected to a LED controller (M00647125, Thorlabs) in a humidified atmosphere (37 °C, 5% CO_2_), following an illumination protocol consisting of consecutive rounds of on and off exposure (2 h per round for the 3 × conditions and 3 h per round for the 2 × conditions).

### Cell culture and seeding

Normal human dermal fibroblasts, nhDF (Lonza, CC-2511), or vimentin depleted human dermal fibroblasts (Vim^−/−^ hDF) were grown in Dulbecco’s Medium Eagle’s Medium (DMEM 41966–029, Thermofisher) supplemented with 10% fetal bovine serum (FBS; 758,093, Serana) and 1% penicillin–streptomycin (15,140,163, Gibco). For the generation of the Vim^−/−^ hDF, primary hDFs (ATCC, PCS-201–010) were immortalized as previously described [75]. The immortalization was confirmed by western blotting using SV40 T large antigen. Vim^−/−^ hDFs were created using CRISPR genome engineering by deleting a defined 488 bp region in the vimentin-encoding gene using one guide RNA (GeneScript, 5′- TGGAAACATCCACATCGATT-3′). Single cell sorting was performed with SH800 Cell Sorter (Sony). Subsequently, the cells were sent for sequencing to confirm the absence of the vimentin gene. Cells were cultured in T-25 cell flasks at 37 °C in a humidified atmosphere with 5% CO_2_. Prior to cell seeding, hydrogel constructs were sterilized by 20 min UV exposure (λ = 254 nm) and coated with 10 µg/mL fibronectin in PBS (bovine plasma fibronectin, 1 mg/mL, 33,010–018, Invitrogen). Fibroblasts were trypsinized using 0.05% trypsin/ethylenediaminetetraacetic acid (EDTA) with phenol red (25,300,054, Gibco). A total of 22,000 cells were seeded on each construct. After 10-h adhesion, topographical patterns were induced as described. All experiments were performed with cells up to passage 15. Every experiment was performed 2 or 3 independent times, with 2 technical replicates in every experimental run.

### Pharmacological treatment

After 5 h of cell adhesion, medium was carefully removed and replaced with medium containing 0.5 µM trichostatin A (HY-15144, Bio-Connect, in PBS), 0.2 µM cytochalasin D (1233, Tocris Bioscience, in DMSO), 10 µM JIB-04 (199,596–05–09, MedChemExpress, in DMSO), PBS or DMSO (276,855, Merck Life Science). Actuation was started 30 min after medium change.

### Immunofluorescence assay

Cells were fixed with 3.7% paraformaldehyde (formalin 37%; 104,033.1000, Merck) for 15 min at room temperature, washed with PBS, permeabilized for 30 min with 0.5% Triton-X-100 (108,643, Merck) in PBS, and blocked for 15 min with 4% goat serum in 0.05% PBS-Tween. Samples were labeled with the primary antibodies rabbit anti-AcH3 (1:300, 06–599, Sigma-Aldrich), rabbit anti-H3K9me3 (1:300, ab8898, Abcam), or mouse anti-γH2AX (1:300, ab22551, Abcam) and incubated overnight at 4 °C. After washing with PBS-Tween, samples were incubated with Phalloidin-Atto 590 (1:70, 93,042, Sigma-Aldrich) and/or the secondary antibodies goat anti-rabbit-Alexa 647 (1:1000, A21244, Molecular Probes) or goat anti-mouse-Alexa 647 (1:200, A21240, Molecular Probes) for 1 h at room temperature. After washing with PBS, nuclei were stained using 4′,6-diamidino-2-phenylindole dihydrochloride (1:200, D9542, Sigma-Aldrich) and stored in the dark at 4 °C until imaging.

### Image acquisition and experimental data analysis

Stained hydrogel constructs were imaged using a Leica TCS SP5 confocal microscope with a 63 ×, 1.2 NA water-immersion objective and 0.25 µm Z-stacks, or with a 20 ×, 0.7 NA objective and 1 µm Z-stacks. Laser intensities and image settings were kept the same across all samples. The signal of the protonated merocyanine was acquired by excitation at λ = 488 nm and detection of the emission at λ = 501–570 nm. CellProfiler [76] pipelines were used to measure nucleus area, solidity (calculated as ObjectArea/ConvexHullArea, where an irregular shape is < 1), and form factor (calculated as 4*π*Area/Perimeter^2^, where 1 is a perfect circle). AcH3 and H3K9me3 levels were quantified as mean intensity values in CellProfiler. Nucleus height was quantified using ImageJ using orthogonal projections of z-stacks [77]. Nuclei were assigned to be above dynamic or static hydrogel regions by using a previously developed MATLAB script [24].

### Optical profilometry

An optical surface profiler (Sensofar Plµ 2300, 20 ×/0.45 NA Nikon objective) was used to measure height profiles. Measurements were processed with PLu Optical Imaging Profiler software (version 2.41) and plotted in GraphPad Prism 10.

### YAP-TAZ localization

nhDF were seeded on hydrogels as described above. After 2 rounds of topographical changes (3 h each), cells were fixed as described previously. Next, YAP was immunolabeled with the primary antibody mouse anti-YAP (1:250, Santa Cruz—101,199) and secondary antibody goat anti-mouse-Alexa 647 (1:500, A21236, Molecular Probes). Actin was stained with Phalloidin-Atto 590 (1:70, 93,042, Sigma-Aldrich), and nuclei were stained with 4′,6-diamidino-2-phenylindole dihydrochloride (1:200, D9542, Sigma-Aldrich). Constructs were image with a Leica TCS SP5 confocal microscope with a 63 ×, 1.2 NA water-immersion objective and 0.25 µm Z-stacks. For quantifying the YAP fluorescence intensity ratio between cytoplasm and nuclei, the cell area was identified based on F-actin staining in the IdentifySecondaryObjects module of CellProfiler, and the cytoplasm was determined via the IdentifyTertiaryObjects module, which removes the nuclear object from the cell area. The CalculateMath modules were used to calculate the YAP Nuc/Cyt ratio, by dividing the measured intensity of YAP of the nucleus by the measured intensity YAP in the cytoplasm.

### Etoposide treatment

nhDF were seeded on 10 µg/mL fibronectin-coated glass coverslips (d = 10 mm), and after 48 h 20 µM etoposide in DMSO (E1383, Sigma-Aldrich) was added. After 24 h of incubation, cells were fixed with 3.7% paraformaldehyde (formalin 37%; 104,033.1000, Merck) for 15 min at room temperature. Cells were immunolabeled for γH2Ax as described above.

### Western blot

Cells (300 000) were washed with ice-cold PBS and lysed with 2 × sample buffer (BioRad, 1,610,737). The lysates were heated for 10 min at 98 °C. The samples were separated by SDS-PAGE using 4–20% acrylamide gradient gels (BioRad), transferred to nitrocellulose membranes (Amersham, 10,600,016) by wet transfer, and blocked for 1 h with 5% milk in Tris buffered saline (TBS) or PBS 0.3% Tween20 at room temperature. The membranes were incubated with primary antibodies anti-SV40 T large antigen (sc-25326, Santa Cruz, 1:1000), anti-vimentin V9 (V6630, Sigma, 1:1000), anti-HSC70 (ADI-SPA-815F, Enzo Life Sciences, 1:1000) diluted in TBS 0.3% Tween20, 3% BSA, 0.02% sodium azide overnight at 4 °C. The membranes were washed for 20 min with TBS 0.3% Tween20 and incubated for 1 h at room temperature with the appropriate HRP-linked secondary antibody (Promega, diluted 1:10 000 in TBS 0.3% Tween20 5% milk). The signal was detected with enhanced chemiluminescence (Amersham, RPN2106) using an iBright machine (CL1000, Thermo Fisher Scientific).

### Quantification and statistical analysis

Data are plotted as mean with standard deviation (SD), unless mentioned otherwise in the figure caption. Statistical analyses were performed using GraphPad Prism 10. Data were tested for normality using the Shapiro–Wilk test. To compare multiple experimental conditions with each other, one-way ANOVA was performed with Tukey’s post hoc analysis for normally distributed data and Mann–Whitney test for nonnormal distributed data. Differences were considered to be significant for *p* values ≤ 0.05.

Graphical representations were made with BioRender.com.

## Supplementary Information

Additional file 1: Figures S1–S14. Fig. S1 Surface profile of dynamic hydrogel with 90 µm-wide grooves. Black profile indicates the mean, SD depicted in gray (*n* = 3). Fig. S2 Representative confocal microscopy images of fibroblast nuclei after 2 or 3 rounds of topographical changes (90 µm-wide grooves) or on flat gels with light exposure (Flat + L). Merge: DAPI-stained nuclei (blue) and acetylation signal (red). AcH3 = histone acetylation. Scale bar = 100 µm. Fig. S3 Representative confocal microscopy images of fibroblast nuclei after 2 or 3 rounds of topographical changes (90 µm-wide grooves) or on flat gels with light exposure (Flat + L). Merge: DAPI-stained nuclei (blue) and methylation signal (red). H3K9me3 = histone trimethylation. Scale bar = 100 µm. Fig. S4 Influence of dynamic topographical changes on YAP/TAZ localization. A) Confocal image of fibroblasts on a stiff hydrogel (E = 350 kPa). Nucleus (blue), F-actin (green), YAP (red), scale bar = 100 μm. B) Fibroblasts on a flat gel, or on a gel with 2 rounds of topographical changes (90 µm-wide pits). Top panel: YAP (gray). Bottom panel: Nucleus (blue), F-actin (green), YAP (red), scale bar = 10 μm. C) Quantification of YAP nuclear localization (Nuc/Cyt ratio) of dynamic conditions for 2 rounds of topographical changes (90 µm-wide pits). Kruskal–Wallis test with a Dunn’s post test, based on *n* ≥ 29 nuclei, each condition performed in duplo with 2 technical replicates every experimental round. Data are represented as mean ± SD. Fig. S5 Addition of 0.5 µM trichostatin A (TSA) increases AcH3 signal in fibroblasts. A) Representative confocal immunofluorescence microscopy images. Blue: DAPI, red: AcH3. Scale bar is 50 µm. B) Quantification of AcH3 intensity levels, *n* ≥ 9 cells. Unpaired *t*-test with *p* < 0.0001. Fig. S6 Detection of double-stranded DNA breaks (γH2Ax) upon addition of chemical compounds (0.5 µM trichostatin A (TSA), 10 µM JIB-04, or 20 µM etoposide (positive control)) using immunofluorescence microscopy. Blue: DAPI, purple: γH2Ax. Scale bar is 50 µm. Fig. S7 Addition of 10 µM JIB-04 increases H3K9me3 signal in fibroblasts. A) Representative confocal immunofluorescence microscopy images. Blue: DAPI, red: H3K9me3. Scale bar is 50 µm. B) Quantification of H3K9me3 intensity levels, *n* ≥ 36 cells. Mann–Whitney test with *p* < 0.0001. Fig. S8 Representative image of vimentin distribution in fibroblasts, cultured on hydrogels with dynamic topographies (2 rounds of 5 h with 90 µm-wide grooves) or flat hydrogels. Blue: nucleus (DAPI), green: F-actin (phalloidin), purple: static, flat regions hydrogel, red: anti-vimentin. Arrows indicate vimentin expression at the cell periphery, while asterisks indicate the distribution of vimentin around the nucleus. Scale bar is 100 µm. Fig. S9 Expression levels of the indicated proteins were determined for fibroblasts (immortalized wildtype, WT, or vimentin knock-out, KO) by western blotting using protein-specific antibodies (3 independent experiments). Fig. S10 Comparison between normal human dermal fibroblasts (nhDF) and immortalized fibroblasts (imm.) used for the generation of Vim^−/−^ cells. A) Representative immunofluorescence images of fibroblasts after 24 h on fibronectin-coated glass. Blue: nucleus, green: F-actin, scale bar is 100 µm. B) Quantification of nuclear parameters: area (i), solidity (ii), and form factor (iii), Mann–Whitney test with *n* ≥ 93 cells and *p* = 0.0478. C) Quantification of AcH3 mean intensity levels, Mann–Whitney test with *n* ≥ 74 cells and *p* = 0.0001. D) Quantification of H3k9me3 mean intensity levels, Mann–Whitney test with *n* ≥ 71 cells. Fig. S11 Dynamic topographies affect nucleus morphology and histone modifications in immortalized dermal fibroblasts. A) Quantification of nucleus morphology: nucleus area (i), solidity (ii), and form factor (iii) after 3 × rounds of topographical changes (flat to 90 µm-wide grooves). Data are represented in violin plots, with dashed lines indicating the median and dotted lines indicating the quartiles. + L = flat gel (not photoresponsive) with light exposure (control). Mann–Whitney test with **** *p* < 0.0001, *** *p* = 0.0003, * *p* = 0.0226, *n* = 176 cells (control) and *n* = 271 cells (dynamic). B) Percentage of nuclei located on the flat (static) part of the hydrogel. Data are represented in a box-and-whiskers plot showing the median, minimum value, maximum value, and the 25th and 75th percentiles. Unpaired *t*-test, *p* = 0.0040, compared to a random distribution of nuclei. *n* = 4 hydrogels with at least 3 processed field of views per gel and ≥ 24 cells per field of view. C) Quantification of mean histone acetylation (AcH3) intensity levels in response to dynamic topographies or flat + light (+ L) control conditions; a.u. arbitrary units. *n* ≥ 88 cells per condition. D) Quantification of mean histone trimethylation (H3K9me3) intensity levels in response to dynamic topographies. *n* ≥ 88 cells per condition. Mann–Whitney test with ** *p* = 0.0029, ns, not significant, *p* > 0.05. Fig. S12 Presence of histone modifications in Vim^−/−^ fibroblasts on dynamic topographies (2 rounds, 90 µm grooves). Blue: DAPI, red: AcH3 or H3K9me3. Scale bar is 50 µm. Fig. S13 Addition of 0.2 µM cytochalasin D (CytD) increases AcH3 signal in fibroblasts after 2 rounds of topographical changes, as detected using confocal immunofluorescence microscopy. Vehicle is DMSO. Blue: DAPI, red: AcH3. Scale bar is 50 µm. Fig. S14 Addition of 0.2 µM cytochalasin D (CytD) increases H3K9me3 signal in fibroblasts after 2 rounds of topographical changes, as detected using confocal immunofluorescence microscopy. Vehicle is DMSO. Blue: DAPI, red: H3K9me3. Scale bar is 50 µm.

Additional file 2: Figure S1. Fig. S1 Expression levels of the indicated proteins were determined for fibroblasts (immortalized wildtype, WT, or vimentin knock-out, KO), by western blotting using protein-specific antibodies (3 independent cell lysates). Figure is showing the uncropped blot.

## Data Availability

Data is provided within the manuscript or supplementary information files.

## Abbreviations

2D: Two-dimensional
AcH3: Acetylation of histone H3
CytD: Cytochalasin D
DAPI: 4′,6-Diamidino-2-phenylindole
DMSO: Dimethyl sulfoxide
DNA: Deoxyribonucleic acid
Dyn: Dynamic
ECM: Extracellular matrix
EDTA: Ethylenediaminetetraacetic acid
Flat + L: Flat gel (not photoresponsive) with light exposure
H3K27me3: Trimethylation of histone H3 at residue K27
H3K9me3: Trimethylation of histone H3 at residue K9
HDAC: Histone deacetylase
HDM: Histone demethylase
Imm: Immortalized
KO: Knock-out
LINC: Linker of Nucleoskeleton and Cytoskeleton
nhDF: Normal human dermal fibroblast
PBS: Phosphate buffered saline
SD: Standard deviation
TBS: Tris buffered saline
TSA: Trichostatin A
Vim: Vimentin intermediate filament
