# Supplementary material for: Dynamic substrate topographies drive actin- and vimentin-mediated nuclear mechanoprotection events in human fibroblasts
Source: BMC Biol. 2025 Apr 7;23:94. doi: 10.1186/s12915-025-02199-7 (PMC11974106; doi:10.1186/s12915-025-02199-7)
Supplement: Supplementary file 1 — Additional file 1: Figures S1–S14. Fig. S1 Surface profile of dynamic hydrogel with 90 µm-wide grooves. Black profile indicates the mean, SD depicted in gray (n = 3). Fig. S2 Representative confocal microscopy images of fibroblast nuclei after 2 or 3 rounds of topographical changes (90 µm-wide grooves) or on flat gels with light exposure (Flat + L). Merge: DAPI-stained nuclei (blue) and acetylation signal (red). AcH3 = histone acetylation. Scale bar = 100 µm. Fig. S3 Representative confocal microscopy images of fibroblast nuclei after 2 or 3 rounds of topographical changes (90 µm-wide grooves) or on flat gels with light exposure (Flat + L). Merge: DAPI-stained nuclei (blue) and methylation signal (red). H3K9me3 = histone trimethylation. Scale bar = 100 µm. Fig. S4 Influence of dynamic topographical changes on YAP/TAZ localization. A) Confocal image of fibroblasts on a stiff hydrogel (E = 350 kPa). Nucleus (blue), F-actin (green), YAP (red), scale bar = 100 μm. B) Fibroblasts on a flat gel, or on a gel with 2 rounds of topographical changes (90 µm-wide pits). Top panel: YAP (gray). Bottom panel: Nucleus (blue), F-actin (green), YAP (red), scale bar = 10 μm. C) Quantification of YAP nuclear localization (Nuc/Cyt ratio) of dynamic conditions for 2 rounds of topographical changes (90 µm-wide pits). Kruskal–Wallis test with a Dunn’s post test, based on n ≥ 29 nuclei, each condition performed in duplo with 2 technical replicates every experimental round. Data are represented as mean ± SD. Fig. S5 Addition of 0.5 µM trichostatin A (TSA) increases AcH3 signal in fibroblasts. A) Representative confocal immunofluorescence microscopy images. Blue: DAPI, red: AcH3. Scale bar is 50 µm. B) Quantification of AcH3 intensity levels, n ≥ 9 cells. Unpaired t-test with p < 0.0001. Fig. S6 Detection of double-stranded DNA breaks (γH2Ax) upon addition of chemical compounds (0.5 µM trichostatin A (TSA), 10 µM JIB-04, or 20 µM etoposide (positive control)) using immunofluorescence microsco [file 12915_2025_2199_MOESM1_ESM.docx]

**Additional File 1**

**Supplementary Information**

**for**

**Dynamic substrate topographies drive actin- and vimentin-mediated nuclear mechanoprotection events in human fibroblasts**

Maaike Bril,^1,2^ Jules N. Boesveld,^1,2^ Leila S. Coelho-Rato,^3^ Cecilia M. Sahlgren,^1,3^ Carlijn V. C. Bouten,^1,2^ Nicholas A. Kurniawan^1,2,^*

1 Department of Biomedical Engineering, Eindhoven University of Technology, PO Box 513 5600 MB Eindhoven, The Netherlands

2 Institute for Complex Molecular Systems, Eindhoven University of Technology, PO Box 513 5600 MB Eindhoven, The Netherlands

3 Faculty of Science and Engineering, Åbo Akademi University, FI-20520 Turku, Finland

* Corresponding author, email: n.a.kurniawan@tue.nl

**Supplementary figures: Figures S1–S14**


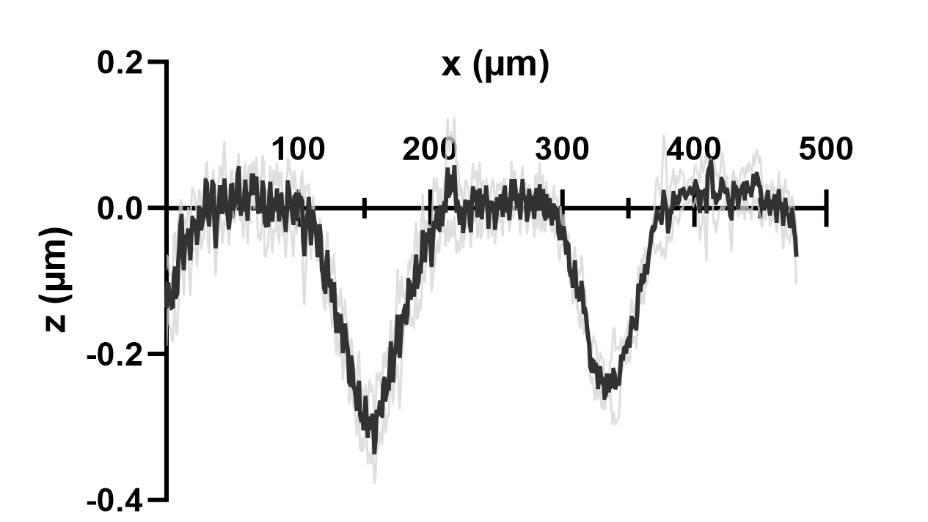


**Figure S1**: Surface profile of dynamic hydrogel with 90 µm wide grooves. Black profile indicates the mean, SD depicted in grey (*n* = 3).

**
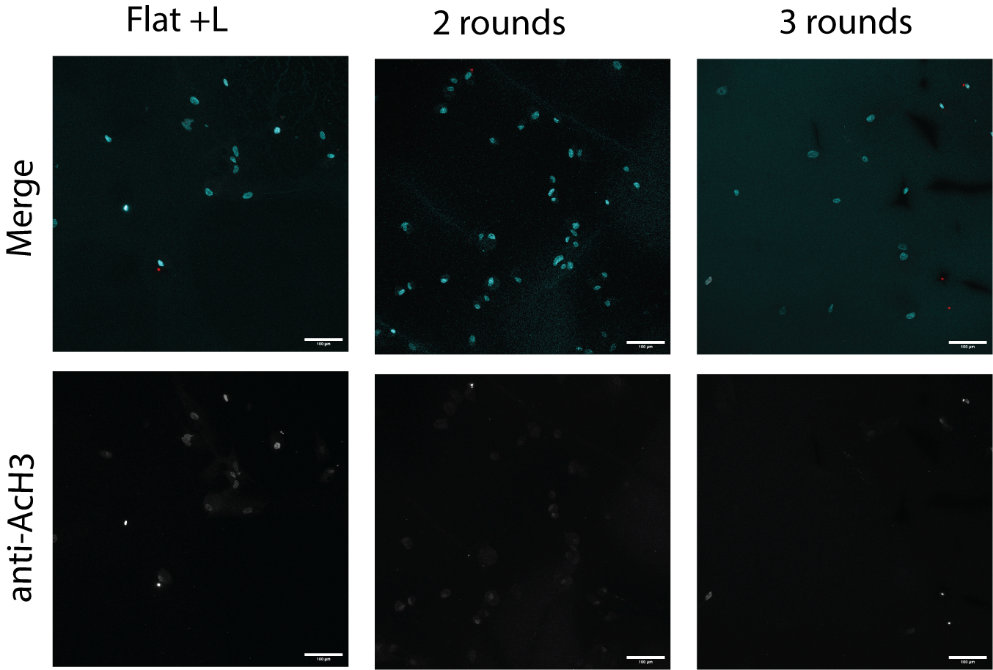
**

**Figure S2**: Representative confocal microscopy images of fibroblast nuclei after 2 or 3 rounds of topographical changes (90 µm-wide grooves) or on flat gels with light exposure (Flat +L). Merge: DAPI-stained nuclei (blue) and acetylation signal (red). AcH3 = histone acetylation. Scale bar = 100 µm.

**
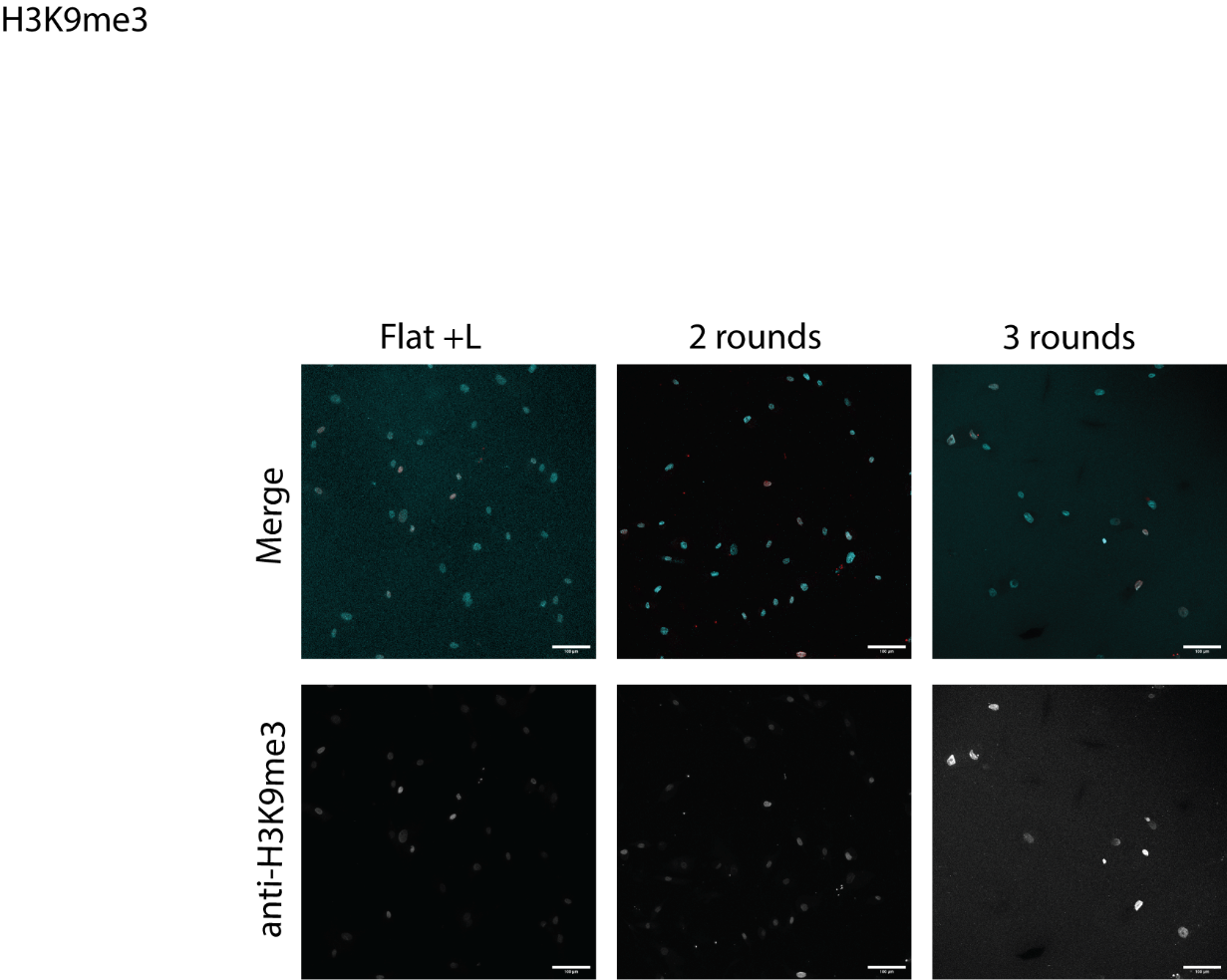
**

**Figure S3**: Representative confocal microscopy images of fibroblast nuclei after 2 or 3 rounds of topographical changes (90 µm-wide grooves) or on flat gels with light exposure (Flat +L). Merge: DAPI-stained nuclei (blue) and methylation signal (red). H3K9me3 = histone trimethylation. Scale bar = 100 µm.


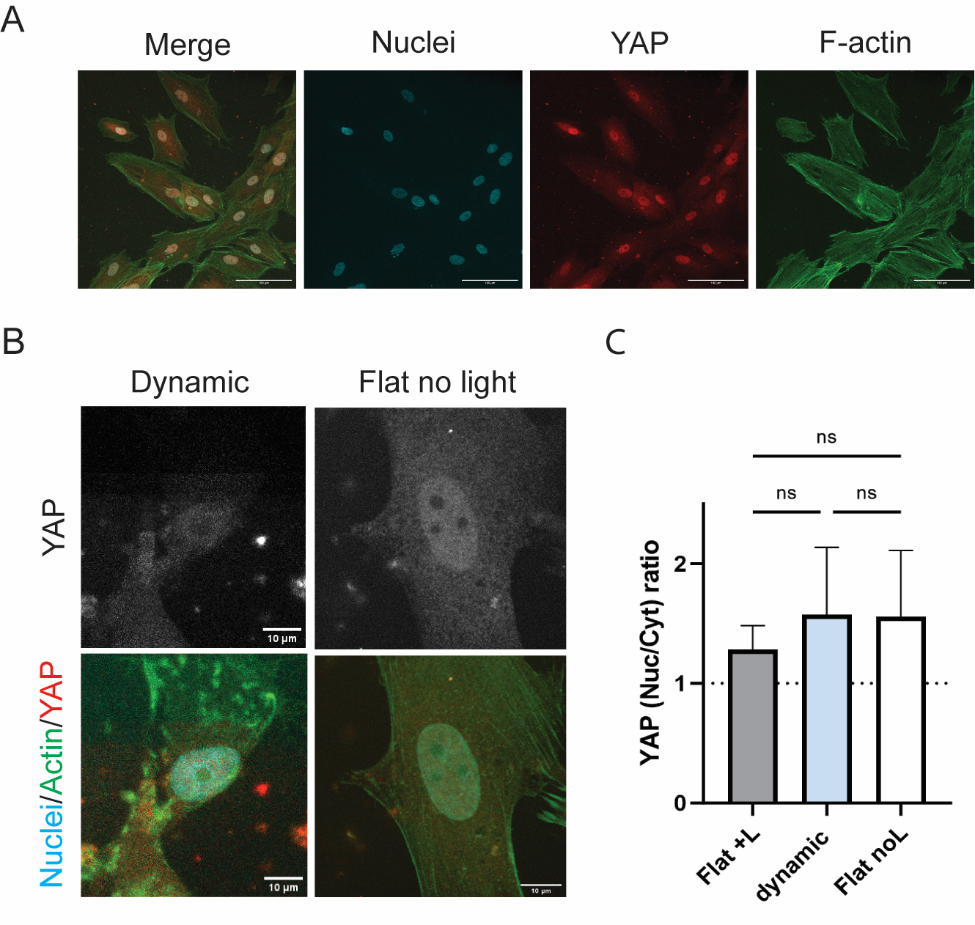


**Figure S4**: Influence of dynamic topographical changes on YAP/TAZ localization. A) Confocal image of fibroblasts on a stiff hydrogel (E = 350 kPa). Nucleus (blue), F-actin (green), YAP (red), scalebar = 100 μm. B) Fibroblasts on a flat gel, or on a gel with 2 rounds of topographical changes (90 µm-wide pits). Top panel: YAP (grey). Bottom panel: Nucleus (blue), F-actin (green), YAP (red), scalebar = 10 μm. C) Quantification of YAP nuclear localization (Nuc/Cyt) ratio) of dynamic conditions for 2 rounds of topographical changes (90 µm-wide pits). Kruskal-Wallis test with a Dunn’s post test, based on *n* ≥ 29 nuclei, each condition performed *in duplo* with 2 technical replicates every experimental round. Data are represented as mean ± SD.


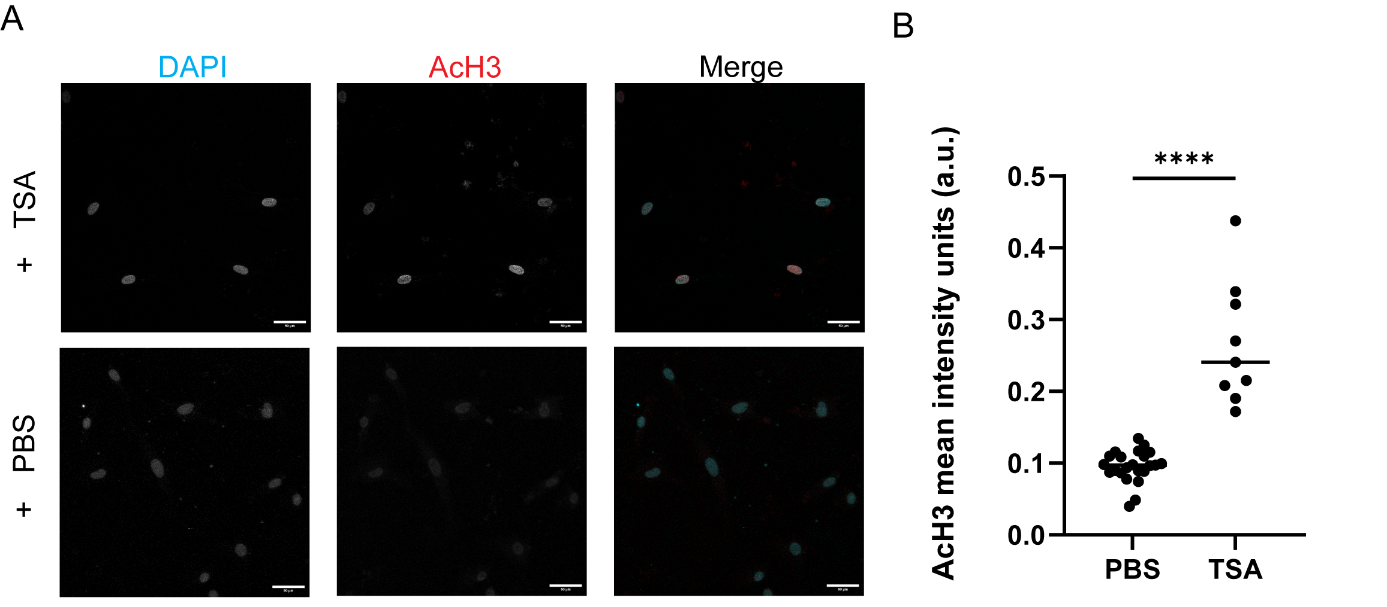


**Figure S5**: Addition of 0.5 µM Trichostatin A (TSA) increases AcH3 signal in fibroblasts. A) Representative confocal immunofluorescence microscopy images. Blue: DAPI, red: AcH3. Scale bar is 50 µm. B) Quantification of AcH3 intensity levels, n ≥ 9 cells. Unpaired t-test with *p* < 0.0001.

**
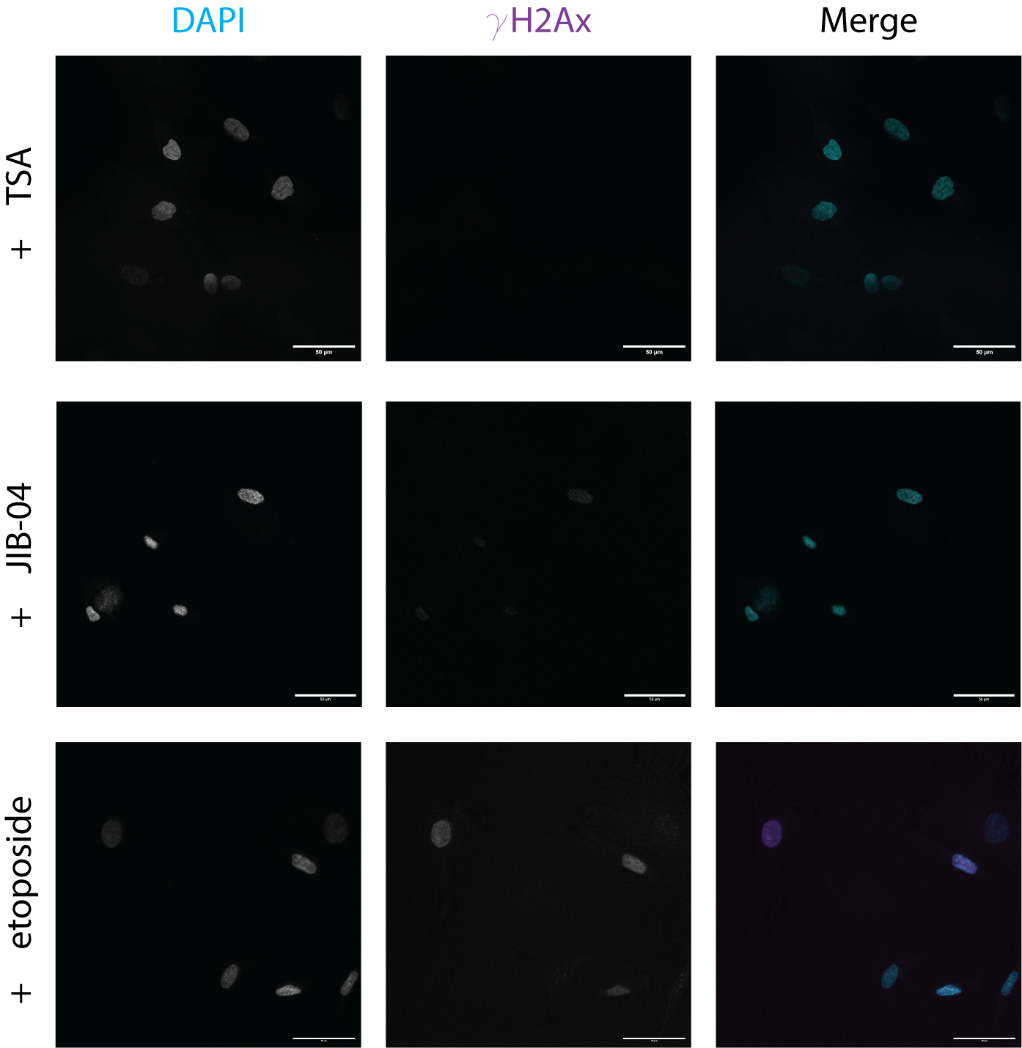
**

**Figure S6**: Detection of double-stranded DNA breaks (γH2Ax) upon addition of chemical compounds (0.5 µM Trichostatin A (TSA), 10 µM JIB-04, or 20 µM etoposide (positive control)) using immunofluorescence microscopy. Blue: DAPI, purple: γH2Ax. Scale bar is 50 µm.


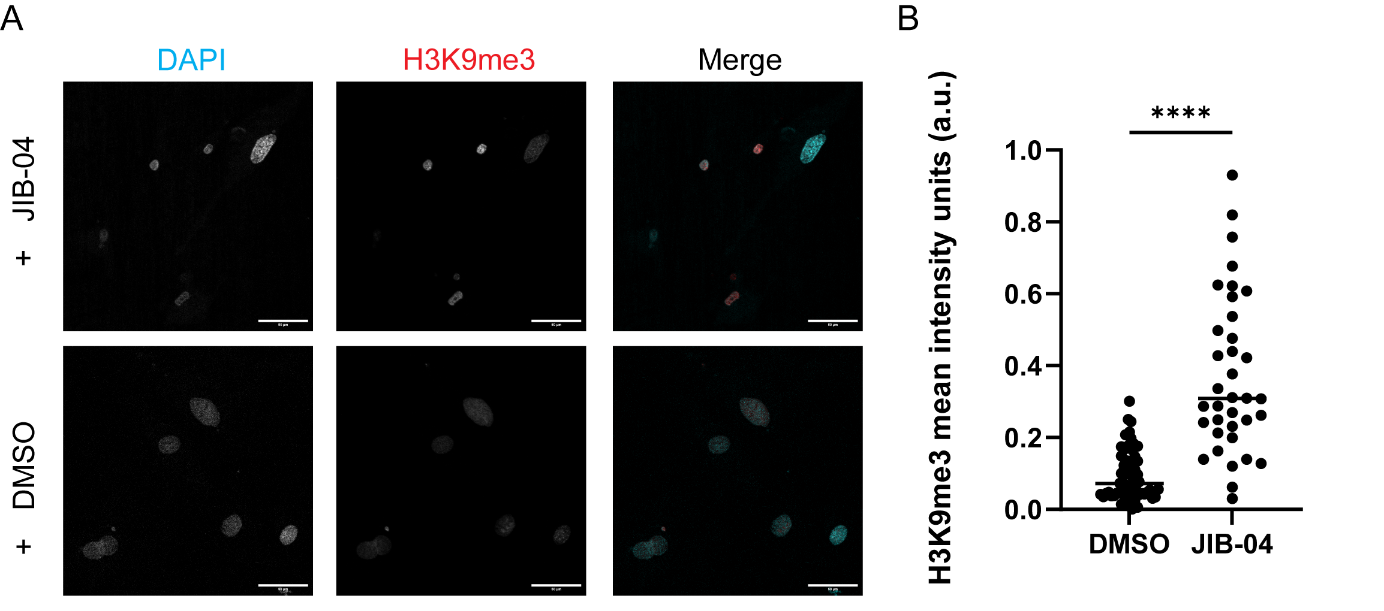


**Figure S7**: Addition of 10 µM JIB-04 increases H3K9me3 signal in fibroblasts. A) Representative confocal immunofluorescence microscopy images. Blue: DAPI, red: H3K9me3. Scale bar is 50 µm. B) Quantification of H3K9me3 intensity levels, n ≥ 36 cells. Mann Whitney test with *p* < 0.0001.


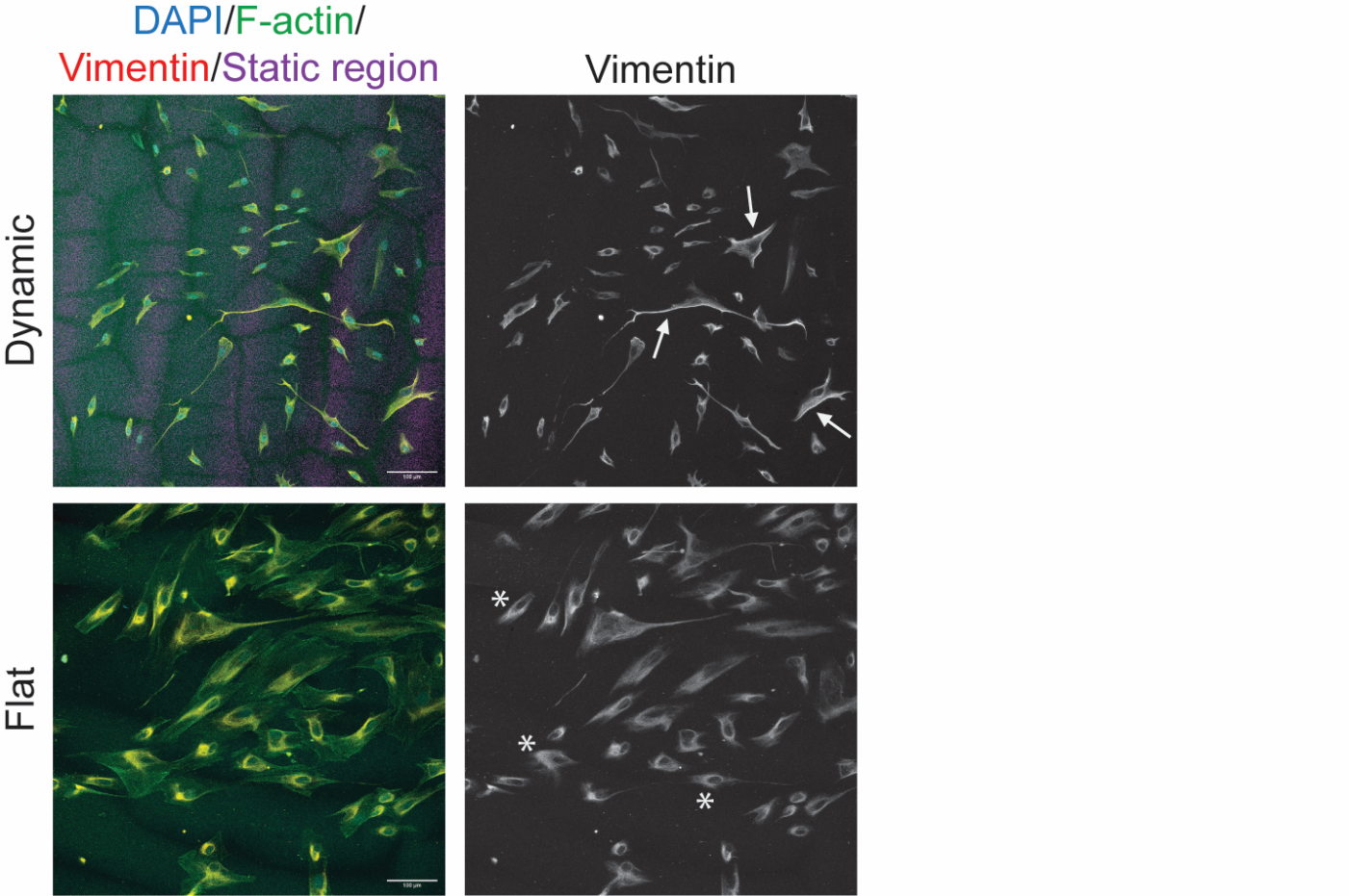


**Figure S8**: Representative image of vimentin distribution in fibroblasts, cultured on hydrogels with dynamic topographies (2 rounds of 5h with 90-µm wide grooves) or flat hydrogels. Blue: nucleus (DAPI), green: F-actin (Phalloidin), purple: static, flat regions hydrogel, red: anti-vimentin. Arrows indicate vimentin expression at the cell periphery, while asterixis indicate the distribution of vimentin around the nucleus. Scale bar is 100 µm.


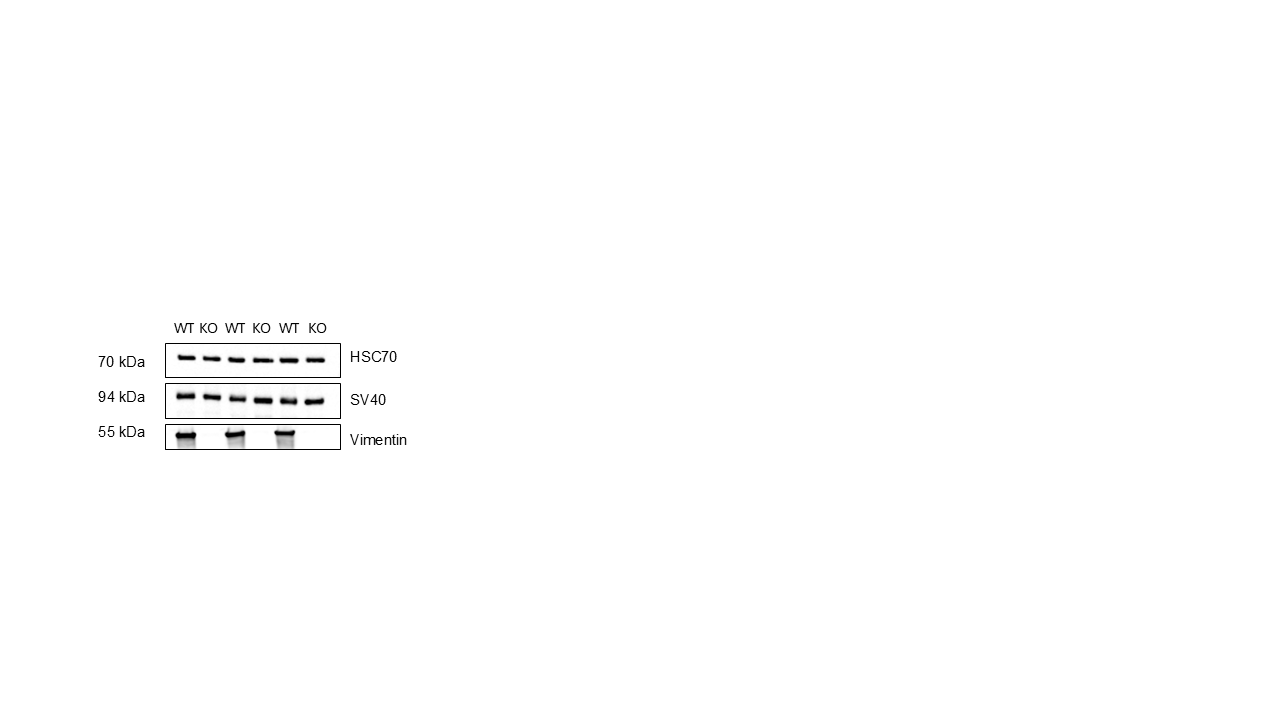


**Figure S9**: Expression levels of the indicated proteins were determined for fibroblasts (immortalized wildtype, WT, or vimentin knock-out, KO) by western blotting using protein-specific antibodies (3 independent experiments).


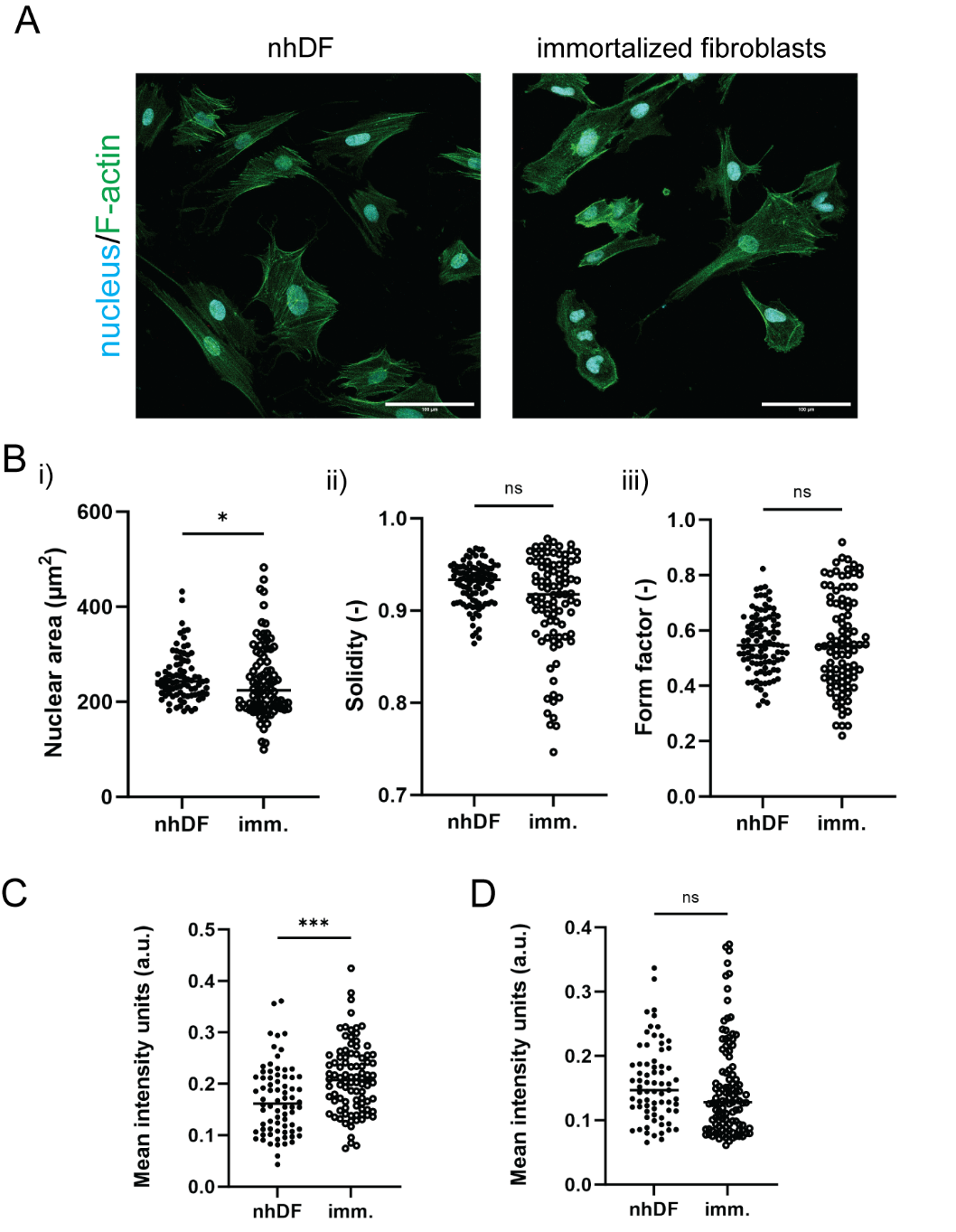


Figure S10 : Comparison between normal human dermal fibroblasts (nhDF) and immortalized fibroblasts (imm.) used for the generation of Vim^-/-^ cells. A) Representative immunofluorescence images of fibroblasts after 24 h on fibronectin-coated glass. Blue: nucleus, green: F-actin, scalebar is 100 µm. B) Quantification of nuclear parameters: area (i), solidity (ii), and form factor (iii), Mann-Whitney test with *n* ≥ 93 cells and *p* = 0.0478. C) Quantification of AcH3 mean intensity levels, Mann-Whitney test with *n* ≥ 74 cells and *p* = 0.0001. D) Quantification of H3k9me3 mean intensity levels, Mann-Whitney test with n ≥ 71 cells.


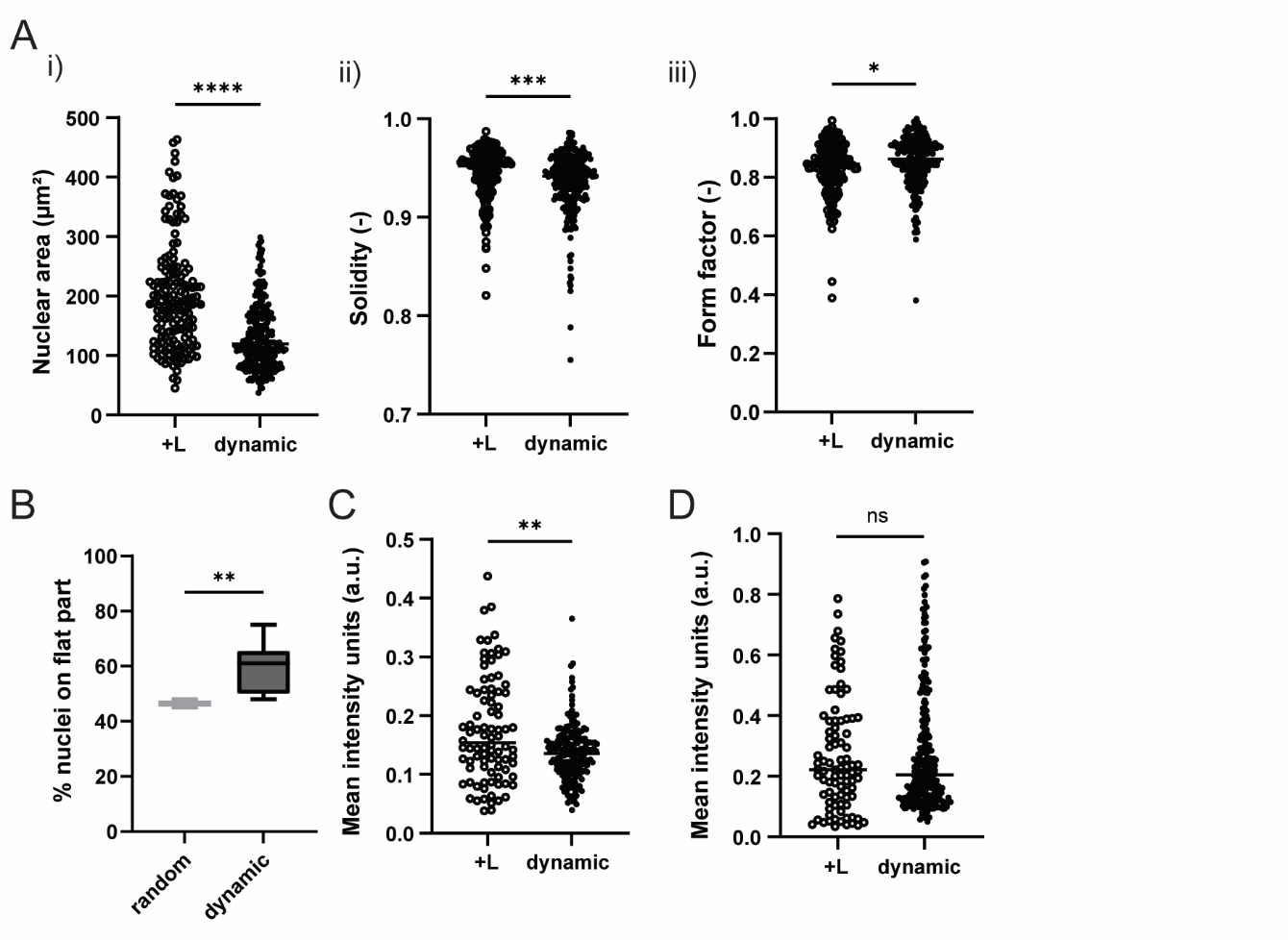
Figure S11: Dynamic topographies affect nucleus morphology and histone modifications in immortalized dermal fibroblasts. A) Quantification of nucleus morphology: nucleus area (i), solidity (ii), and form factor (iii) after 3× rounds of topographical changes (flat to 90 µm-wide grooves). Data are represented in violin plots, with dashed lines indicating the median and dotted lines indicating the quartiles. +L = flat gel (not photoresponsive) with light exposure (control). Mann-Whitney test with **** p < 0.0001, *** p = 0.0003, * p = 0.0226, *n* = 176 cells (control) and *n =* 271 cells (dynamic). B) Percentage of nuclei located on the flat (static) part of the hydrogel. Data are represented in a box-and-whiskers plot showing the median, minimum value, maximum value, and the 25^th^ and 75^th^ percentiles. Unpaired t-test, *p* = 0.0040, compared to a random distribution of nuclei. *n* = 4 hydrogels with at least 3 processed field of views per gel and ≥ 24 cells per field of view. C) Quantification of mean histone acetylation (AcH3) intensity levels in response to dynamic topographies or flat + light (+L) control conditions; a.u. arbitrary units. *n* ≥ 88 cells per condition. D) Quantification of mean histone trimethylation (H3K9me3) intensity levels in response to dynamic topographies. *n* ≥ 88 cells per condition. Mann-Whitney test with ** *p* = 0.0029, ns, not significant, *p* > 0.05.

**
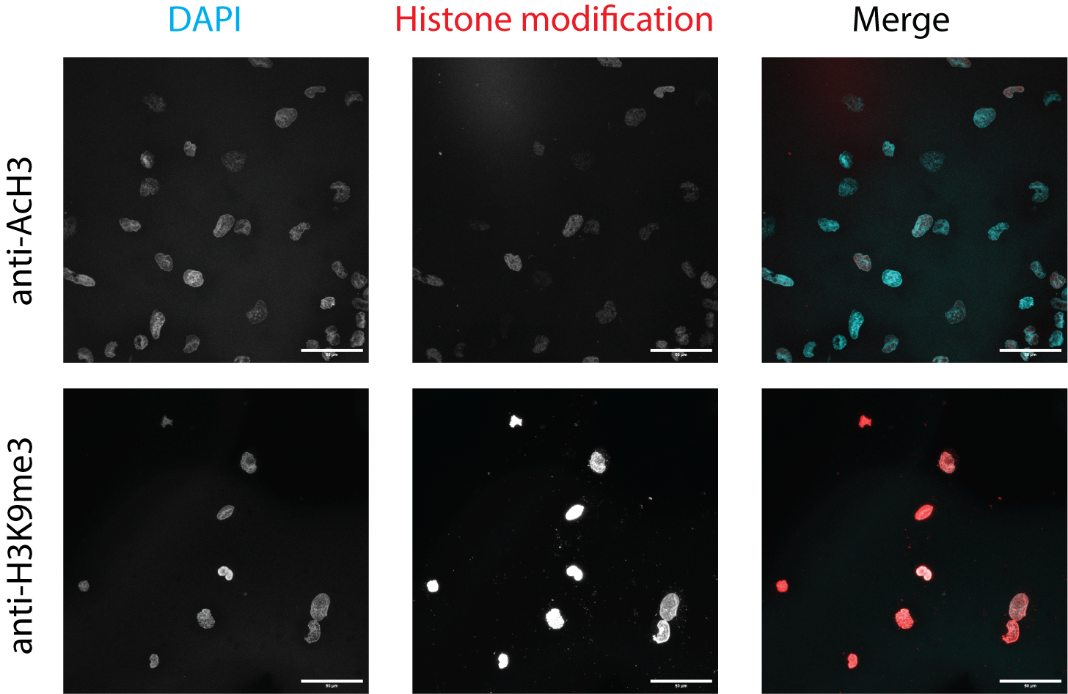
**

**Figure S12**: Presence of histone modifications in Vim^-/-^ fibroblasts on dynamic topographies (2 rounds, 90 µm grooves). Blue: DAPI, red: AcH3 or H3K9me3. Scale bar is 50 µm.


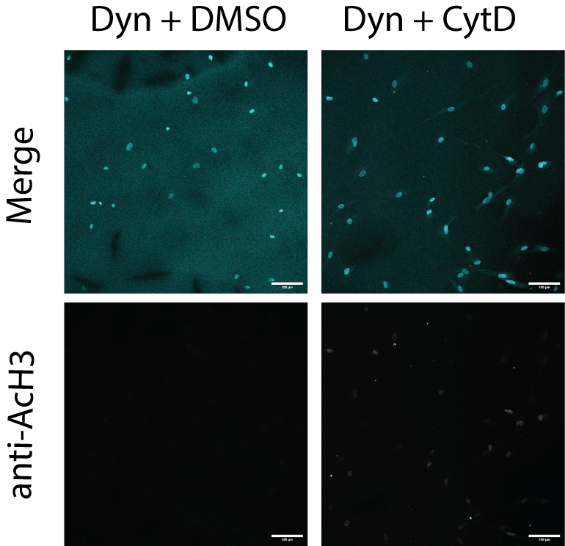


**Figure S13**: Addition of 0.2 µM Cytochalasin D (CytD) increases AcH3 signal in fibroblasts after 2 rounds of topographical changes, as detected using confocal immunofluorescence microscopy. Vehicle is DMSO. Blue: DAPI, red: AcH3. Scale bar is 50 µm.


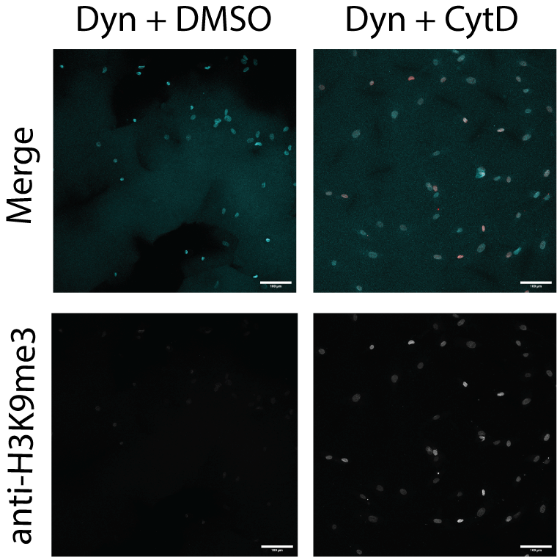


**Figure S14**: Addition of 0.2 µM Cytochalasin D (CytD) increases H3K9me3 signal in fibroblasts after 2 rounds of topographical changes, as detected using confocal immunofluorescence microscopy. Vehicle is DMSO. Blue: DAPI, red: H3K9me3. Scale bar is 50 µm.
