# Supplementary material for: Dynamic substrate topographies drive actin- and vimentin-mediated nuclear mechanoprotection events in human fibroblasts
Source: BMC Biol. 2025 Apr 7;23:94. doi: 10.1186/s12915-025-02199-7 (PMC11974106; doi:10.1186/s12915-025-02199-7)
Supplement: Supplementary file 2 — Additional file 2: Figure S1. Fig. S1 Expression levels of the indicated proteins were determined for fibroblasts (immortalized wildtype, WT, or vimentin knock-out, KO), by western blotting using protein-specific antibodies (3 independent cell lysates). Figure is showing the uncropped blot. [file 12915_2025_2199_MOESM2_ESM.docx]

**Additional File 2**


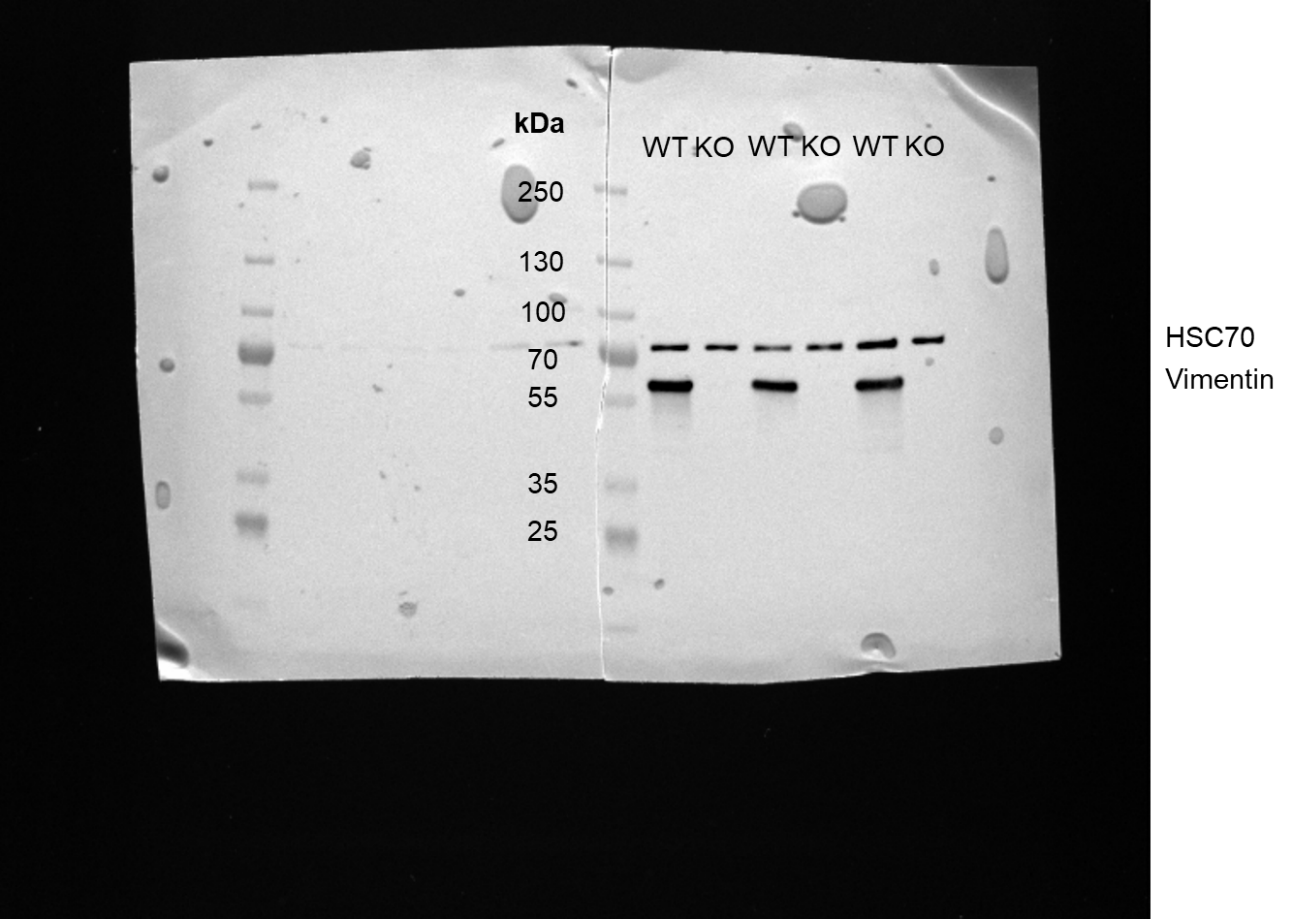


Figure S1: Expression levels of the indicated proteins were determined for fibroblasts (immortalized wildtype, WT, or vimentin knock-out, KO, by western blotting using protein-specific antibodies (3 independent cell lysates). Figure is showing the uncropped blot.
